# Assessment of the Blood Separation Performances of Asymmetric Cellulose Acetate Membranes Prepared through Combined Vapor-Induced Phase Separation and Electrospinning

**DOI:** 10.1021/acsami.5c05283

**Published:** 2025-05-20

**Authors:** Gian Vincent Dizon, Yu-Jen Huang, Fang-Cheng Lin, Irish Valerie Maggay, Yung Chang, Antoine Venault

**Affiliations:** R&D Center for Membrane Technology and Department of Chemical Engineering, 34900 Chung Yuan Christian University, Chung-Li 32023, Taiwan (R.O.C)

**Keywords:** cellulose acetate VIPS membrane, electrospinning, asymmetric membrane, blood filtration, cell-free plasma

## Abstract

This study introduces the formation of a composite cellulose acetate (CA) membrane for the removal of blood cells from blood-derived solutions. A vapor-induced phase separation (VIPS) CA membrane formed the bottom layer of the composite membrane. The exposure time to vapors was adjusted with the aim to open the pores of the surface exposed to the nonsolvent and obtain a membrane with a mean pore size of less than 0.4 μm. A 20 min exposure led to few pores in the microfiltration range decorating the top surface, while much more numerous pores were observed on the bottom surface in contact with the substrate during the phase-inversion process. Differences between the top and bottom surface porosity in turn influenced wetting by water. The membranes with fastest top and bottom surfaces wettability were selected. Then, nanofibers with a mean pore size of 2.8 ± 0.3 μm were formed by electrospinning a CA solution on the bottom surface of the VIPS membrane. It resulted in the formation of a composite membrane with an asymmetric structure (large top surface pores and smaller bulk and bottom surface pores). The membranes were proven to be nonhemolytic (hemolysis rate <2%) and showed a plasma clotting time in the range 16–22 min. Applied to the gravity-driven filtration of platelet-poor plasma, platelet-rich plasma, and 10-fold and 5-fold dilutions of whole blood, complete blood count analyses showed that the optimized membrane referred to as 15V-15E could separate all platelets, red blood cells, and white blood cells from plasma (100% removal in each case). In addition, the hemolysis of the filtrate obtained with membrane 15V-15E after separation of the 5-fold dilution was extremely low. While the electrospun layer proved efficient for the gradual removal of most cells, SEM and confocal images also highlighted the efficacy of the VIPS layer to reject cells that managed to penetrate deeper in the bulk. However, a VIPS membrane alone failed (no flow) justifying the need for the composite structure. Overall, these membranes show significant potential for facilitating the separation of cells from plasma, which could be applied to the detection of biomarkers in plasma.

## Graphical abstract

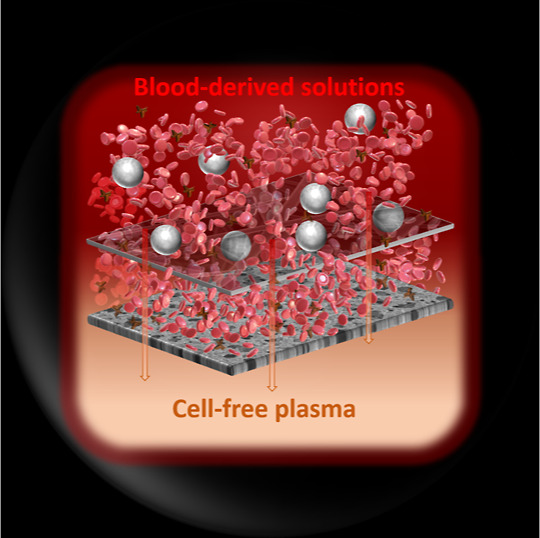

## Introduction

The vapor-induced phase separation (VIPS) process, first mentioned in 1918 by Zsigmondy and Bachmann,^1^ is a process in which penetrations of nonsolvent from a vapor phase into a liquid polymer/solvent phase trigger phase inversion leading to membrane fabrication. Because the nonsolvent initially exists in a gaseous state, the gas/liquid interface naturally creates an initial resistance to mass transfer. This leads to a slower phase inversion process in comparison to the so-called nonsolvent-induced phase separation (NIPS or wet-immersion) method, where the nonsolvent is already in liquid form. When applied to the same polymer/solvent system, VIPS and NIPS yield distinct morphologies and, therefore, potential applications. For instance, when using polyvinylidene difluoride and common solvents like *N*-methylpyrrolidone or dimethylacetamide, VIPS with water vapors results in the formation of porous top surfaces and symmetric cross sections.^2^ In contrast, NIPS with liquid water leads to the development of a top-skin layer with small dispersed pores and a cross section decorated with well-connected macrovoids.^2^ As highlighted in the recent review of Ismail et al.,^3^ hydrophobic polymers such as PVDF, polysulfone, and poly­(ether sulfone) account for the majority of materials used in the preparation of VIPS membranes.

VIPS applied to hydrophilic cellulose acetate (CA) has rarely been employed. Nevertheless, some groups have reported this material/process combination for various applications, although CA was often supplemented with other polymers or pore-forming additives. Chen et al. blended CA with polyethylenimine, then cross-linked by polyisocyanate, and formed microfiltration membranes for adsorbing Cu^2+^ and bovine serum albumin (BSA).^4^ Moghiseh et al. examined the NIPS/VIPS process combination to prepare porous CA membranes, also utilizing polyvinylpyrrolidone or polyethylene glycol as pore-forming additives.^5^ They highlighted the membranes’ potential for water treatment in biomedical applications. In their study, Ammendola et al. explored the use of VIPS for crafting CA microcapsules, with a focus on encapsulating fragrances.^6^ After a storage period of one year, the encapsulated fragrances exhibited no loss in their potency, suggesting that the protective CA shell surrounding the fragrance was effectively dense. More recently, Marino et al. mentioned the use of VIPS for the formation of optically transparent porous cellulose acetate scaffolds employed as blood–brain barrier models.^7^ They exposed CA/acetone films to vapors of water and ethanol for 6 days, resulting in the formation of a porous scaffold. More recently, Wang et al. used a method which they referred to as vapor-assisted nonsolvent induced phase separation (VNIPS) in which high solvent evaporation rate (controlled by high temperature) and nonsolvent influx (controlled by the relative humidity) were primarily responsible for the phase inversion of the CA/additive/solvent system before immersion in a nonsolvent.^8^ Finally, Xiong et al. utilized vapors of ethanol to create membranes containing both CA and poly­(styrene-*block*-butadiene-*block*-styrene).^9^

Electrospinning is another method to create porous membranes by utilizing electrical forces to draw a polymer solution into a fine, continuous jet, ultimately resulting in nanoscale or microscale fibers with a high surface area-to-volume ratio. The solvent system, the addition of a cosolvent, the presence of salt, the polymer concentration, and the spinning conditions (flow rate, applied voltage, and working distance) are some of the essential parameters to control in order to adjust the morphology of the fibers.^10^ This process has been employed extensively to create nanofibers from CA or CA derivatives,^11,12^ in particular for biomedical applications,^13–15^ due to the hydrophilicity and relative biocompatibility of CA. While several process parameters generally influence the morphology of polymeric nanofibers (voltage, working distance), Liu and Hsieh’s study highlighted the significance of the solvent system in the production of cellulose acetate (CA) nanofibers.^11^ They found that by mixing specific solvents, such as dimethylacetamide and acetone or acetic acid, they were able to achieve the production of homogeneous nanofibers. This control over the solvent system allowed them to manage viscosity effectively, preventing bead formation at low viscosity (<1.2 poise) and instability during the spinning process at high viscosity (>10.2 poise).^11^

While centrifugation is a conventional method for blood separation, it suffers from several drawbacks such as being time- and energy-consuming, requiring bulky equipment, and posing challenges for integration with detection systems.^16–18^ Alternative systems based on filtration offer potential solutions to these issues, although they demand careful design of the membrane. Membranes applied to the filtration of whole blood and blood-derived products need to possess several key properties associated with the material system and porous morphology. Hydrophilicity is one of these properties, as better wetting characteristics facilitate the flow of plasma, mitigate clotting, and improve the biocompatibility and hemocompatibility of the membrane system.^19–21^ Logically, the porous characteristics (pore size and porosity) are critical parameters that need to be adjusted on the basis of the target of the separation. For instance, the reported pore size for leukoreduction membranes is often very large (several micrometers^22^), that of hemodialysis membranes small (5–10 nm),^23^ while the pore size for membranes applied to the separation of blood cells from plasma should fall in between. In plasmapheresis, the main purpose is to remove the plasma (and keep the cells)^24^ and exchange it with a substitute saline solution. Nevertheless, a similar separation can be conducted aiming to keep the plasma and remove the blood cells for improved clinical diagnosis^17,25^ as plasma contains numerous biomarkers (nucleic acids, proteins, etc.) or pathogens whose detection can be extremely challenging in the presence of cells (cell-membrane rupture interferes with the detection). Membranes applied to blood plasma separation should be hydrophilic and have pore sizes significantly smaller than the dimensions of the smallest cellular component. If we consider platelets, red blood cells, and white blood cells as the main cellular components, then 1 to 2 μm pore size filters should be efficient. However, because cells, in particular red blood cells, are highly deformable to flow through very small capillaries in the human body,^26^ microfiltration membranes (0.1–1 μm) are actually needed to allow plasma to flow through while retaining all cells. A challenge may arise if the pore size is too small, related to the viscosity of blood which not only slows down the transport through the filter but also increases the shear stress.^27^ The latter may then cause cell hemolysis, which must be avoided. In addition, other than preventing hemolysis during the removal of blood cells, the filter must also prevent clotting.^19^ Ahmed and Kaplan discussed some commercial membranes available in the United States for therapeutic plasma exchange, and mentioned a pore size in the range 0.3–0.5 μm, to allow the removal of the smallest cells (platelets).^24^ These commercial filters have a fibrous morphology composed of either polypropylene or polyethylene.

The electrospinning process can create pores several micrometers in diameter. However, the pore size of VIPS membranes is smaller and usually falls in the microfiltration range, although the porous features (pore size, bulk, and surface porosity) also depend on other parameters associated with the formulation (polymer type, molecular weight, and solvent system). From this observation, associating both processes, by electrospinning a CA solution on a CA VIPS membrane, would arise in the formation of an asymmetric porous structure, whose average pore size would be similar to that of the VIPS membrane but with a gradient of pore size in the bulk of the membrane, which could potentially benefit the separation of blood cells from plasma. Ideally, leukocytes would be removed first by the electrospun layer and smaller cells rejected then by the VIPS layer. Gradual screening of cells would enable flow and likely reduce stress, leading to cell lysis.

We propose in this work to test this assumption and spun fibers of cellulose acetate atop a VIPS membrane, which has, to the best of our knowledge, not been reported before. The main objective, as depicted in Figure 1, is to test the feasibility of plasma separation, where blood cells are separated using the generated asymmetric structure. This study begins by (I) discussing the formation and properties of the VIPS layer, followed by (II) the fibrous layer and (III) the composite membrane. The next major part of this study concerns the filtration of blood products and the assessment of the composite membrane performances during the cell removal from platelet-poor plasma (PPP), platelet-rich plasma (PRP), and different dilutions of whole blood. It is believed that this study provides valuable results and directions for the design of effective membranes for the preparation of cell-free plasma.

**Figure 1:**
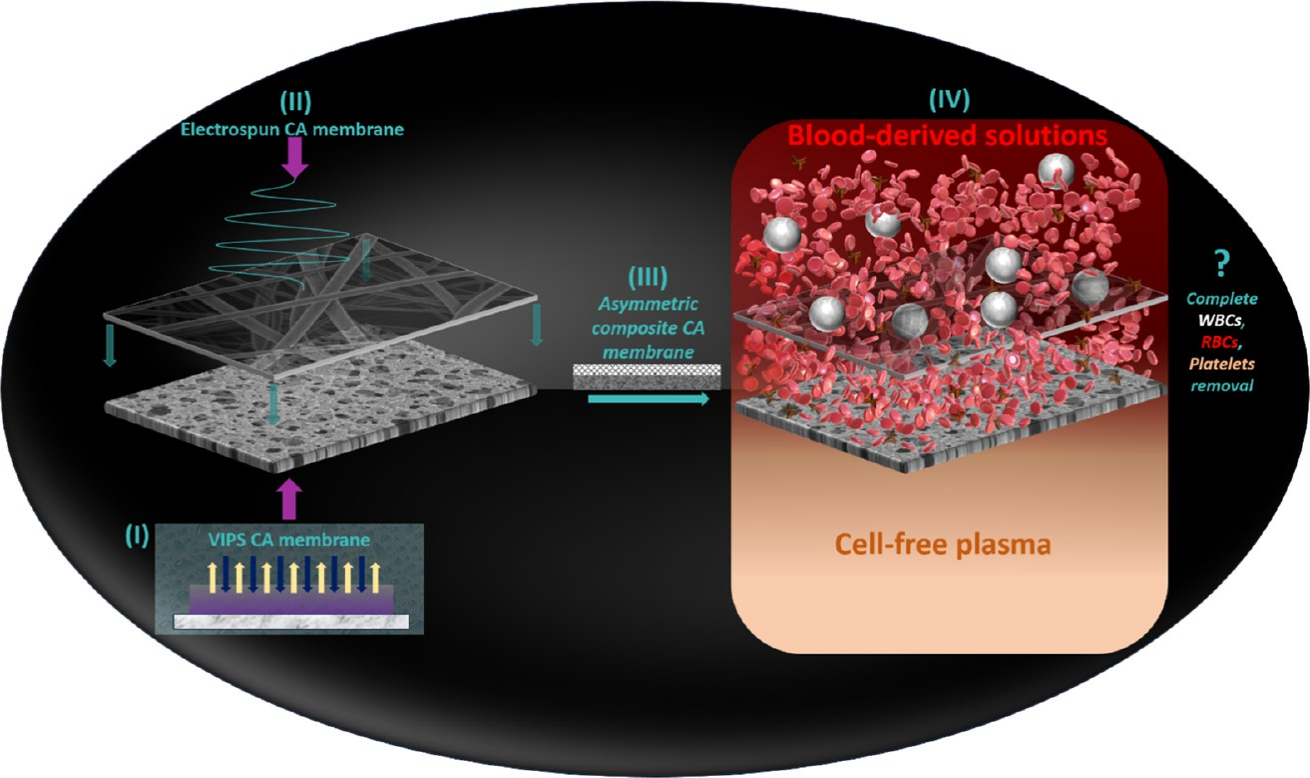
Scheme of the main objectives of this study which involve combining (I) VIPS and (II) electrospinning to create and characterize (III) a CA asymmetric membrane and (IV) evaluate its performance in the removal of cells from different blood-derived solutions.

## Experimental Methods

### Materials

Cellulose acetate was obtained from Eastman. It has an approximate molecular weight of 60,000 g/mol. Dimethylacetamide (DMAc) was purchased from Macron Fine Chemicals. Acetone was obtained from Duksan Pure Chemicals Co., Ltd. DI water was produced with an ELGA (Veolia) lab water purification system. Whole blood was obtained from the Mackay Memorial Hospital (Taipei branch, Taiwan) and initially drawn from a pool of healthy volunteers. Dual-Range Bradford Reagent (1×) was from Visual Protein. Dulbecco’s phosphate-buffered saline (DPBS) was obtained from Sigma-Aldrich and used to incubate the membranes prior to filtration but also to prepare dilutions of whole blood.

### Casting Solution Preparation and Membrane Formation by VIPS

Cellulose acetate polymer was dissolved in DMAc at room temperature with continuous stirring. The polymer content was varied within a range of 10 wt % to 15 wt %. Subsequently, the obtained solution, a glass substrate, and a metallic casting knife were placed inside a sealed chamber. Inside the chamber, humidity and temperature were controlled with values set at 70% humidity and 30 °C, respectively. Approximately one hour after these parameters were adjusted, the solution was applied onto the substrate using the casting knife. The initial thickness of the casting was 300 μm. The system was then exposed to humid vapors for varying durations, ranging from 0 to 20 min. Following this exposure, the systems were removed from the chamber and immersed in a bath of DI water, where phase separation was completed (in cases of short exposure times) or where the membrane was washed. In the cases of shorter exposure times, immersion in a second bath of DI water was used to wash the membranes and remove all traces of solvent. In situations where no exposure to humid vapors occurred (0 min of exposure), the solution was cast in ambient air and immediately immersed in DI water.

### Solution Preparation and Membrane Formation by Electrospinning

Cellulose acetate polymer was dissolved in a mixture of DMAc and acetone (in a 2:1 volume ratio) at room temperature, while continuously stirring for 24 h. The choice of the solvent/cosolvent system was based upon a previous work that mentioned the obtaining of “good fibers” with this system, although it depended also on the concentration of the polymer (hence the viscosity of the system) and on spinning parameters.^28^ Here, the polymer content was varied 12 or 15 wt %. The solutions were transferred to a syringe adapted for an electrospinning machine to prepare nanofibers. Electrospinning was carried out for 2 h at a flow rate of 4.8 mL/h with an applied voltage of 25.5 kV. The working distance was set at 15.24 cm, and the fibers were collected on a rotating drum collector revolving at an angular velocity of 320 rpm.

To prepare asymmetric membranes consisting of a VIPS layer and an electrospun layer, the VIPS membrane was attached to the rotating collector before the electrospinning process. For pure electrospun membranes, an aluminum sheet wrapped around the rotating collector served as the substrate and the membranes were gently removed from it after the electrospinning process.

### Membrane Physical Characterization

The structure of membranes was analyzed using a Hitachi S4800 field emission scanning electron microscope (FE-SEM). Before testing, samples were dried, secured onto an SEM holder with double-sided tape, and then coated with platinum for 150 s. Observations were conducted at an accelerating voltage of 3 kV.

The membrane average bulk pore size was assessed by using capillary flow porometry. A PMI instrument was employed, and samples (VIPS membrane, electrospun membrane, or composite VIPS/electrospun membrane) were wetted with Galwick liquid (PMI). Three independent tests were conducted, and the membrane pore size was determined as the average value ± the standard deviation (SD).

Surface pore size distributions of the VIPS membrane samples were analyzed using ImageJ, a Java-based image processing software. After the scale was established, the image was converted to either 8 bit or 16 bit format, followed by the application of a threshold. To minimize noise, the despeckle function was utilized from the process menu along with the binary options for erosion and dilation to refine the feature outlines. Specific measurements, notably Feret’s diameter, were configured, and the analysis was then conducted. Lastly, a summary report was generated, concentrating on Feret’s diameter parameter with 100 bins.

The membrane porosity was determined by weight measurements. First, dry samples (diameter 1.3 cm) were weighed and then immersed in butanol for 24 h. Then they were removed from the alcohol bath, and the excess alcohol was gently wiped off with tissue cloth. Finally, the samples were weighed again. The porosity could then be determined from the knowledge of the dry and wet weights, along with the density of the alcohol and polymer, according to a formula reported elsewhere.^29^

### Wettability Tests

Water contact angle measurements were conducted using an OCA 15EC contact angle instrument (DataPhysics Instruments). Approximately 4 μL of deionized water was dispensed onto the surface of the dried membranes affixed to a glass slide. The WCA was automatically measured at 0.7 s intervals over a duration of 300 s.

### Permeability Tests

The water permeability of the composite membranes was assessed, and tests were performed using a 2 cm diameter membrane disk placed in a 2.5 cm diameter stainless steel module connected to a pressurized water reservoir. The pressure was set to 1.5 bar for 1 h for compaction. It was then decreased to 1 bar, and the water flux was recorded after reaching a steady state.

### Filtration of Blood Products

In this study, platelet-poor plasma (PPP), platelet-rich plasma (PRP), and diluted whole blood were used as the feed for the filtration tests. PPP was obtained by centrifugation of whole blood at 400*g* for 10 min at 37 °C, followed by a second centrifugation of the supernatant at 800*g* for 10 min at 37 °C. PRP was obtained by centrifugation of whole blood at 180*g* for 5 min at 25 °C. Diluted whole blood was prepared by dilution with DPBS. In this study, both 10-fold and 5-fold dilutions were tested.

Prior to the filtration, the membrane samples (of surface area 4.9 cm^2^) were wetted with 2 mL of ethanol for 2 min and then washed 3 times with DPBS. Then, they were immersed in a fresh solution of DPBS overnight. 3 mL of the blood solution (PPP, PRP, or diluted blood) was placed in a syringe connected to a disposable plastic module fitting 2.5 cm diameter membrane samples. Due to the low volume of blood (and so the low initial head pressure), pressure was gently manually applied with the piston at the very beginning of the test in order to spread the blood solution and wet the membrane with the blood solution. The piston was then immediately removed, and gravity-driven filtration proceeded. The filtrate was then collected in plastic tubes and analyzed with a complete blood count (CBC) analyzer (XN-450, Sysmex). The overall gravity-driven flux could be assessed from the knowledge of the membrane surface area, the feed volume, and the filtration time.

After the filtration tests, the membranes were analyzed by SEM and a confocal microscope. For the SEM analyses, the samples were first gradient dried with PBS/ethanol solutions. For the laser scanning confocal microscopy observations with a Nikon A1R^+^, the samples were first fixed and stained with a 2.5 v % PBS solution of glutaraldehyde for 4 h at 4 °C and then with a 5 mg/mL solution of 4′,6-diamidino-2-phenylindole (DAPI) in PBS for 1 h at 4 °C. Five independent tests were performed for each type of filtration.

### Total Plasma Protein

The total plasma protein concentration was measured for 5-fold and 10-fold diluted whole blood and their corresponding filtrates. Bradford protein assay was used to determine the total protein concentration of these solutions. The diluted whole blood solutions and their filtrates were further diluted 100-fold in order to fit inside the working range of the Bradford protein assay (20–1000 μg/mL). In a 96-well plate, 10 μL of the samples (diluted whole blood and their filtrates) were added to individual wells. Then 200 μL of the Bradford reagent was added following the incubation of the well plate to 37 °C for 15 min. Then the absorbance of the samples was measured at 595 nm. The total plasma protein concentration was then calculated based on a linear calibration line prepared by measuring 5 different concentrations of diluted whole blood (100×–500×). The presented data were acquired from 4 independent solutions for each condition. For the testing of other biomarkers such as glucose, total cholesterol, and triglycerides, samples were sent to an independent testing center at Yea-Dong Institute of Medical Laboratory located in Taoyuan, Taiwan.

### Hemolysis and Plasma Clotting Time Tests

Hemolysis tests were run using membrane samples or the filtrates after filtration according to the following protocol.

For tests conducted on the membranes, the samples (of diameter 1.3 cm) were each placed into a 15 mL centrifuge tube together with 3 mL of PBS and 60 μL of whole blood resulting in an RBC concentration of 10^8^ cells/mL. The membranes were then incubated with the blood solution at 37 °C for 1 h in a water bath. Following incubation, the tubes were centrifuged at 510*g* for 5 min to let the erythrocytes settle at the bottom of the tube. Hemolysis extent was determined by measuring the absorbance of the supernatant at 542 nm using a microplate reader (BioTek PowerWave XS). Controls were DI water (hemolytic solution, ≈100% hemolysis) and a PBS solution (no hemolysis). The reported data represent the average of 3 independent measurements. The hemolysis of the samples was calculated as shown in eq 1.

1 $$Hemolysis\;\left(\%\right)=\frac{{sample\;OD}_{542}-{PBS\;OD}_{542}}{{DI\;water\;OD}_{542}-{PBS\;OD}_{542}}$$

For tests conducted on the filtrate after the separation of diluted whole blood, the filtrate was kept at 4 °C in a refrigerator for 24 h, leading to partial blood fractioning. Subsequently, the absorbance of 200 μL of the supernatant was measured at 542 nm. The controls used were the supernatant of diluted whole blood before filtration (0% hemolysis) and diluted whole blood in DI water (100% hemolysis).

The nonthrombogenic property of the composite membranes was evaluated by measuring the plasma-clotting time. PPP was first obtained by the centrifugation of whole blood. Membrane samples having a diameter of 0.3 cm were placed on the walls of a 96-well plate together with 160 μL of PPP. The solution was then recalcified by the addition of 40 μL of 0.1 M CaCl_2_ to each well in the well plate. The absorbance at 660 nm of the plasma solution was then recorded every 2 min for 2 h at 37 °C using a microplate reader (BioTek PowerWave XS). The plasma clotting time was obtained as the average of the onset of the clotting process. This was determined as the time when the change in the measured absorbance of the PPP was greater than 0.010. The presented data were obtained from 3 independent samples for each condition of membrane preparation.

## Results and Discussion

### Aspects of VIPS Membrane Fabrication

Cellulose acetate membranes were produced by using the VIPS process, with the initial intention to create porous membranes. The existing body of knowledge on CA membrane formation is limited to a few studies and either concerns blended membranes^4^ or a combination of VIPS and wet immersion.^5^ The effect of the exposure time to nonsolvent vapor on the membrane morphology was scrutinized fixing the polymer concentration to 15 wt %. As the exposure time to vapors increases from 0 to 20 min, the results of Figures 2 and S1 show that the morphology of the membranes undergoes distinct changes. Initially, for very short exposure times (up to 1 min), the membranes exhibit rather dense top and bottom surfaces and large pores similar to macrovoids decorating the cross section. These morphological features (skin layer on top and at the bottom, macrovoids in the cross section) are characteristic from a phase separation process by wet immersion where fast transfers can initially occur due to the presence of a liquid/liquid interface. The time the system stays in the vapor phase is too short to allow for a significant role of the vapors of nonsolvent on the final membrane morphology. With longer exposure times, small pores become noticeable in particular on the bottom surface. On the top surface, they are also created but only noticeable for long exposure times. On the bottom surface, the pore density increases gradually with exposure time. All pores originate from the growth of polymer-lean domains within the chamber. This phenomenon also results in a lacy cross-sectional pattern. However, coalescence of polymer-rich domains (a phenomenon that also depends on the viscoelastic properties of the polymer) may also occur either for long exposure times or high nonsolvent concentration, thereby reducing pore connectivity. It is particularly evident in layers closer to the upper surface which is constantly exposed to a larger concentration in nonsolvent from the beginning of the phase-inversion process. Consequently, in this CA/solvent system, the upper surfaces bear a resemblance to those obtained through wet immersion. A similar phenomenon was previously observed by Chen et al. in their study of CA/cross-linked polyethylenimine membranes.^4^ Under these membrane fabrication conditions, high porosity is retained solely on the lower surface, which means that pore density and size asymmetries were created.

**Figure 2:**
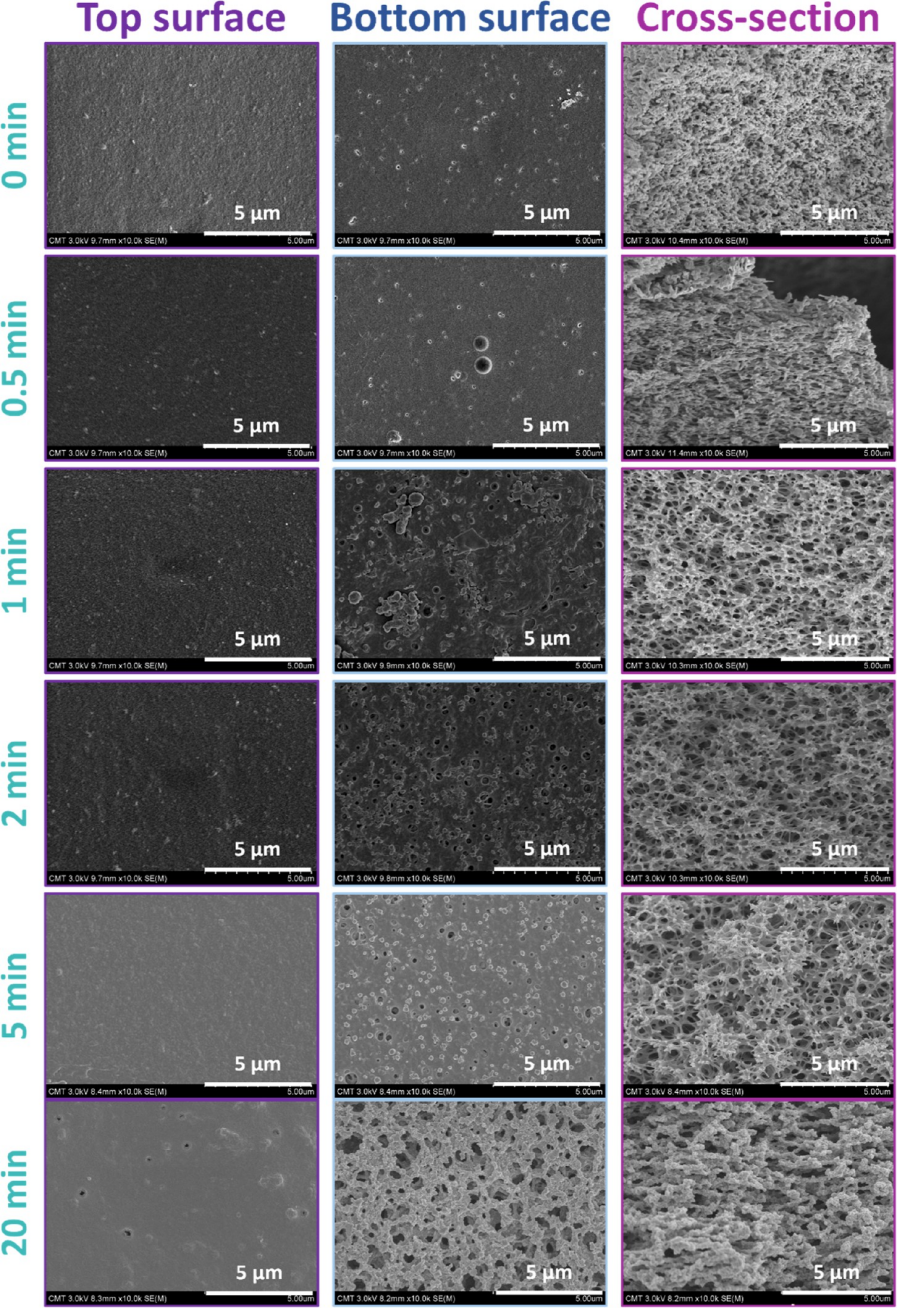
Effect of the exposure time to nonsolvent vapors on the morphology of the VIPS membranes. All images have a magnification of ×10,000.

It is known that a reduction in the polymer concentration in the casting solution arises from the formation of larger pores. As seen in Figure S2, the change in pore size is only visually noticeable from the observation of the bottom surfaces when reducing the polymer concentration from 15 wt % to 12 wt %. Conversely, the top surfaces exhibit a relatively dense morphology, with only a limited number of observable pores. The coalescence of the polymer-rich domains at the interface between the atmosphere of nonsolvent and the nascent film is also facilitated when the polymer concentration decreases (because the viscosity of the system decreases and so weakens the constraint on domain coalescence). This likely explains the quasi-absence of pores on the top surface at 10 wt %. Thus, this membrane was not considered further for the following tests and analyses.

The difference in morphology between the top and the bottom surfaces is evident when analyzing the surface pores of the membranes. Specifically, the surfaces of both analyzed membranes exhibit a striking contrast in the distribution of pores within the MF domain. From the image analysis, membranes prepared with a 12 wt % polymer content possessed up to 160 pores of diameter 0.1 μm. For those prepared from a 15 wt % polymer content, this number was found to be in the same range (Figure S3a,c). In stark contrast, while the top surface of both membranes also showcases pores within the MF range, the count is low, with fewer than 10 pores in each range on every analyzed image (Figure S3b,d). Additionally, the impact of polymer concentration is further underscored by the mean bulk pore size (Figure S3e).

The influence of the fabrication parameters on the membrane surface properties can also be seen through the measurement of the water contact angle in the air of the different surfaces. Since the polymer is the same, alteration in the surface structure is the sole driver influencing the WCA. Several well-known models have been proposed rationalizing the effect of the membrane structure including the Cassie–Baxter equation^30^ or the Wenzel equation.^31^ In the case of absorbing surfaces (porous surfaces made of hydrophilic materials), the WCA is also influenced by the pore size and porosity of the substrate.^32–34^ Figure 3 illustrates that the exposure time to nonsolvent vapors indeed affects membrane wetting, as an extended exposure time correlates with a reduction in WCA, which is associated with an increase in surface porosity. For an exposure to vapors of 20 min, the WCA reaches 0 more rapidly for a 12 wt % concentration than for 15 wt %, aligning with the polymer concentration’s impact on pore size (a lower polymer concentration is associated with larger pores and easier wetting by water of intrinsically hydrophilic surfaces). Furthermore, the findings suggest that the WCA observed on the bottom surfaces (Figure 3c,d) is consistently lower than those recorded on the top surfaces (Figure 3a,b), which is due to the different surface morphologies (see SEM images of the top and bottom surfaces in Figures 2 and S2). Prolonged exposure times lead to the formation of increasingly porous surface structures, resulting in lower WCA and in a more rapid decrease in WCA.

**Figure 3:**
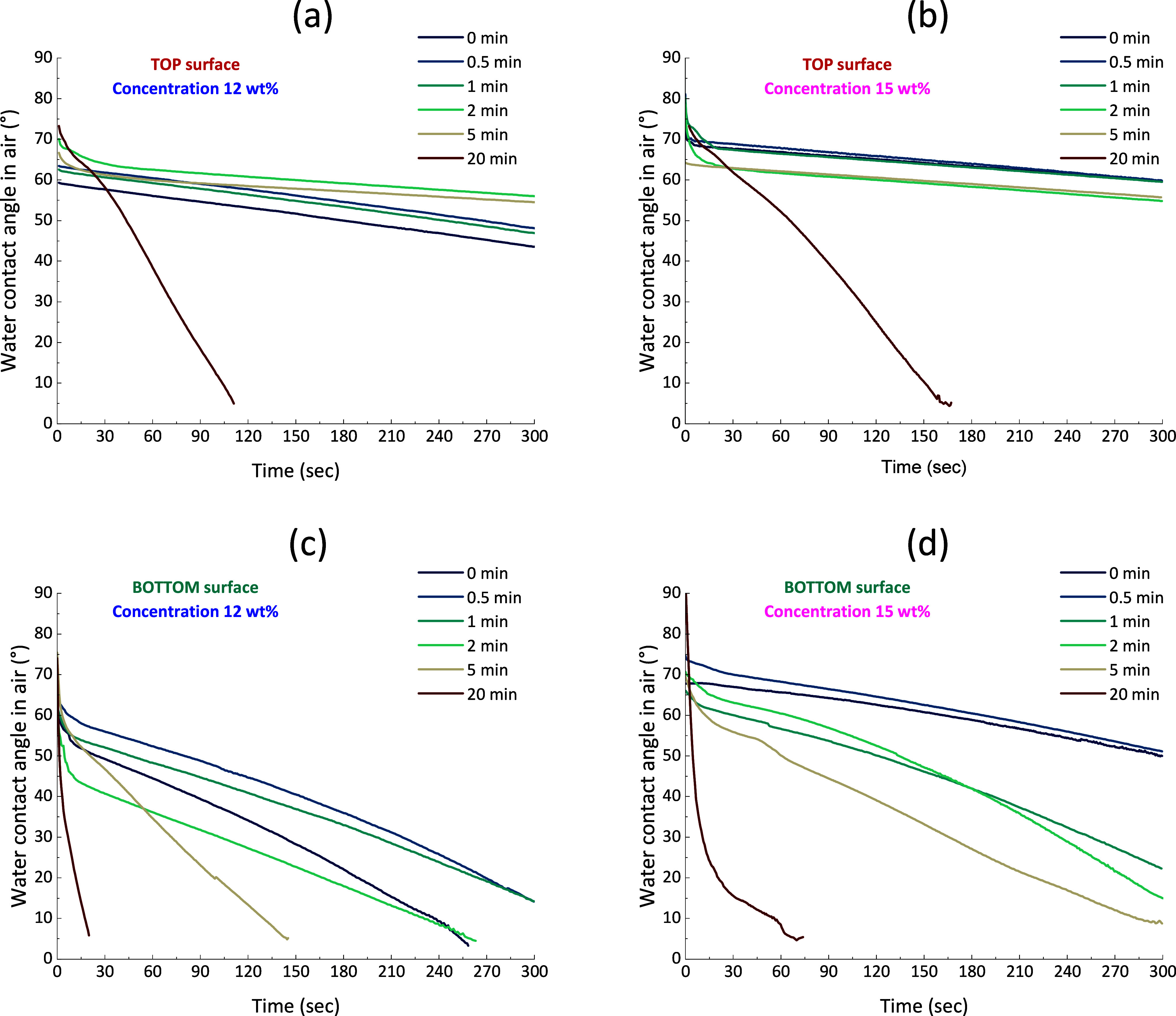
Effect of the fabrication parameters on surface wettability. (a) Top surface, CA concentration 12 wt %; (b) top surface, CA concentration 15 wt %; (c) bottom surface, CA concentration 12 wt %; (d) bottom surface, CA concentration 15 wt %.

### Electrospun Membrane Fabrication

The electrospinning process was chosen to fabricate the top layer of the membrane because large pores are required for the intended application of this layer (removal of larger cells, still enabling flow). As mentioned in the introduction, the existing literature on CA membrane development through this process is extensive.^11,12,15,28^ Therefore, our initial attempts and parameter settings drew from both a prior publication^28^ and our experience with a similar process involving another polymer.^35,36^

The selection of specific parameters, including the polymer concentration (12 or 15 wt %), solvent system (acetone/dimethylacetamide), applied voltage (25.5 kV), feed rate (4.8 mL/h), and distance between the needle and collector (15.2 cm), resulted in the formation of an electrospun membrane characterized by its uniformity, devoid of beads, and possessing pores with a size of 1.2 ± 0.1 μm and 2.8 ± 0.3 μm (Figure 4). In addition, one can visually observe the apparent increase in the fiber diameter with the polymer concentration in the dope, which has been explained before through the change in viscosity,^10^ while the pore size follows an opposite trend. This result has been rationalized earlier and is correlated to the change in fiber diameter: larger-diameter fibers tend to widen the interfiber space.^37^

**Figure 4:**
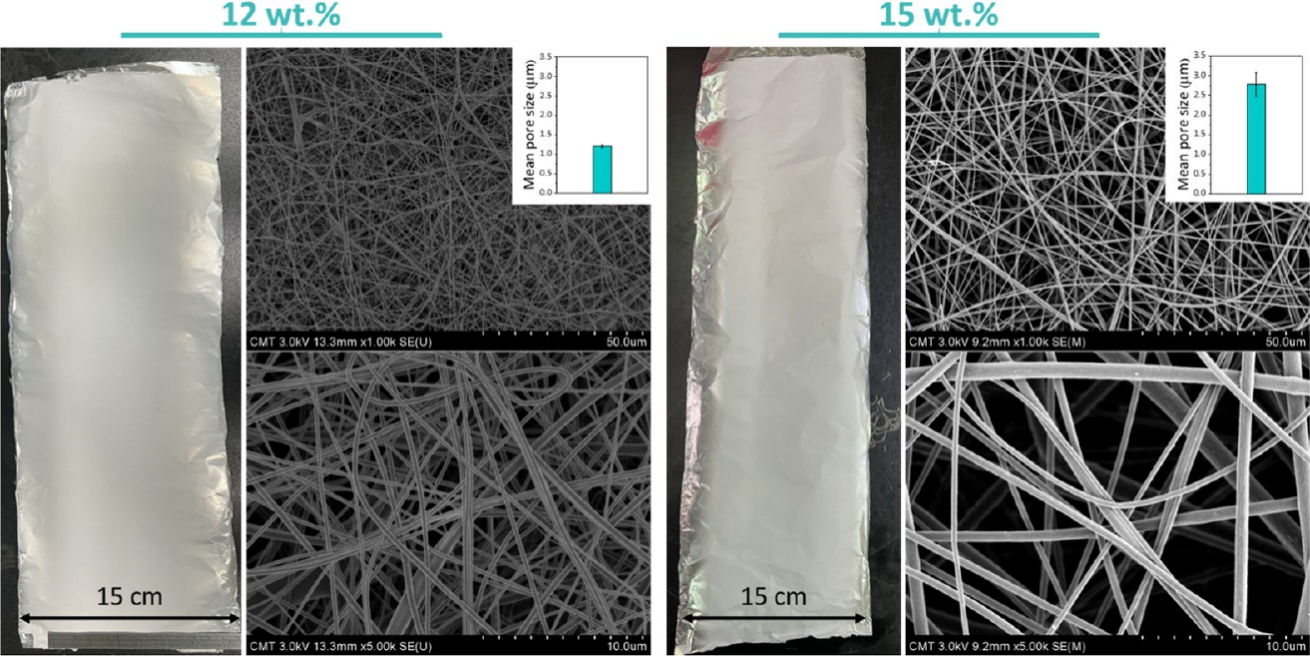
Physical properties of CA electrospun membranes, including photographs, FE-SEM images, and mean pore size. Nanofibers were prepared from dope solution with 12 wt % (left panel) and 15 wt % (right panel) polymer content.

The membrane design aligns well with the intended structure of the overall filter. Considering the viscosity of blood solutions and in order to minimize the resistance to flow in the upper layer, the electrospun membrane with larger pores was selected for the fabrication of the composite filter.

### Characterization of the Composite Membrane

After adjusting the morphology of the VIPS membrane and independently electrospinning a larger-pore membrane, the subsequent step involved directly spinning nanofibers onto the optimized VIPS membrane to obtain an asymmetric membrane. CA solutions with 15 wt % concentration were electrospun onto the bottom surface of either 12 or 15 wt % VIPS membranes. Photographs along with SEM images of the resulting composite membranes are shown in Figure 5. Large surface area membranes could be prepared, which could be an advantage for future scale-up. The top surface of the composite membrane corresponds to the electrospinning layer, while the bottom surface corresponds to the top surface of the VIPS membrane. The asymmetric structure is revealed from the SEM image of the cross section, showing a fibrous mat seating atop the bicontinuous VIPS layer. Since both layers were formed from the same material (CA), the electrospun layer is expected to interact well with the VIPS layer with minimum delamination that would impair the separation performance.

**Figure 5:**
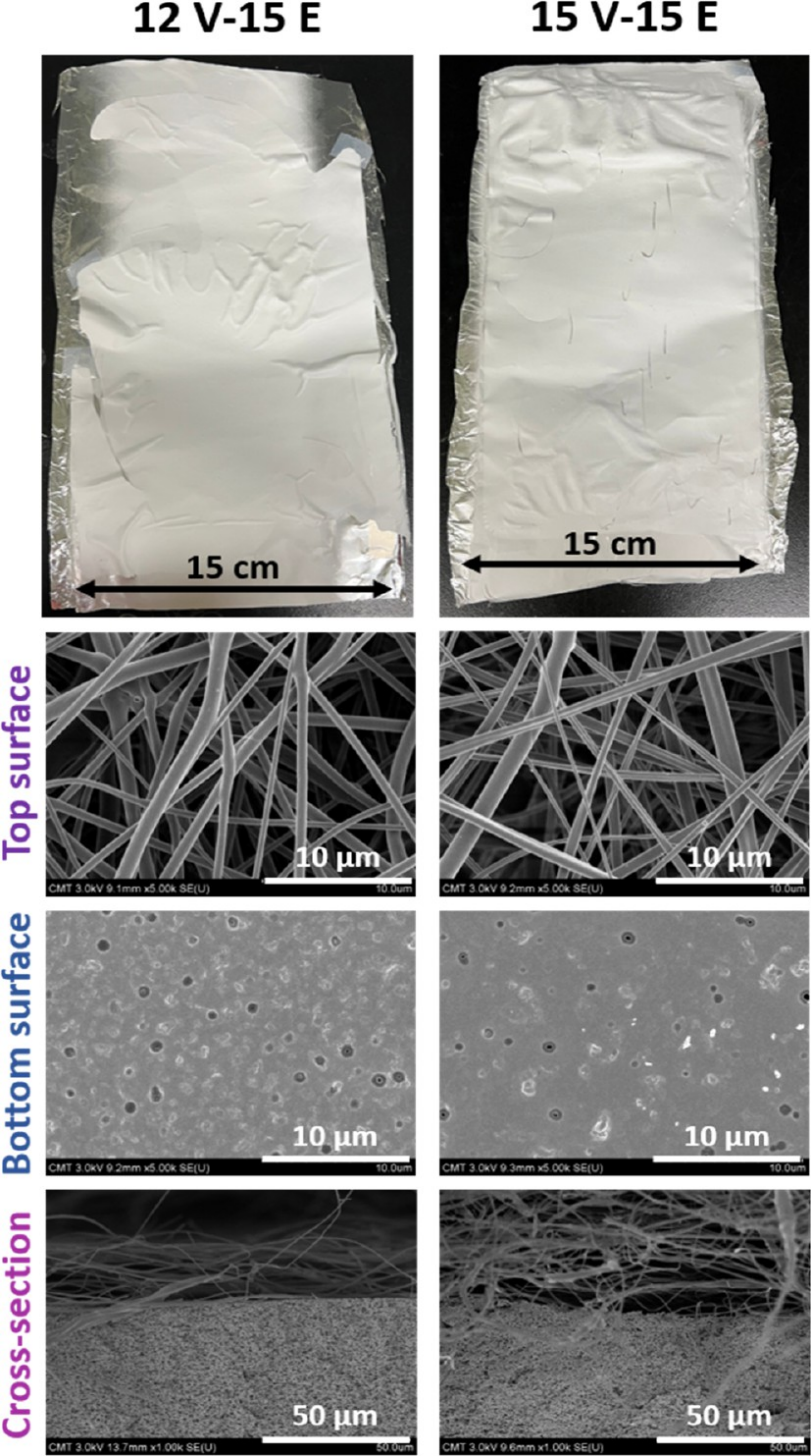
Morphology of the 12V-15E and 15V-15E composite membranes.

The porosity and mean bulk pore size of the membranes are compiled in Figure 6. The composite membranes maintain a high porosity, ranging between 83 and 84%. This value falls between that of the pure VIPS membrane (79–81%) and the pure electrospun membrane (84–88%). The mean bulk pore size of the final system is that of the layer with the smallest mean pore size (VIPS layer). Therefore, samples 12V-15E and 15V-15E exhibit comparable mean pore sizes to samples 12V and 15V.

**Figure 6:**
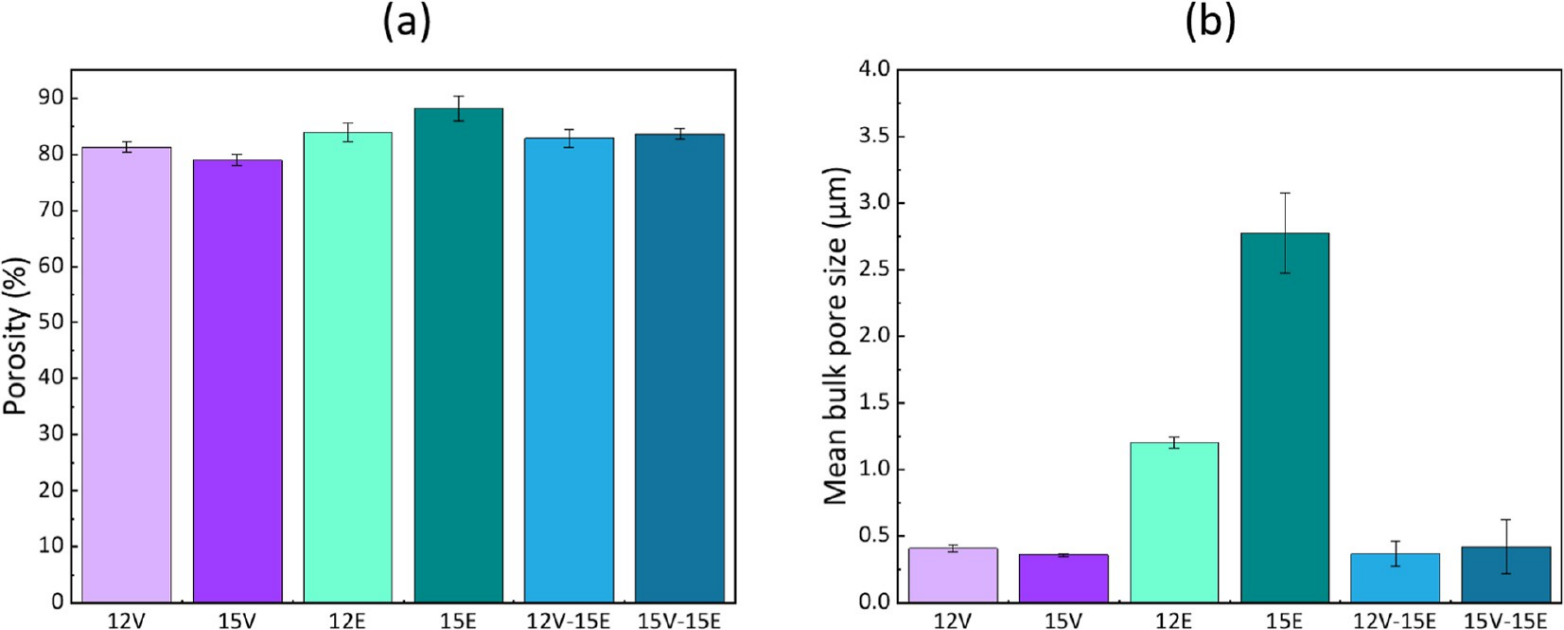
Physical properties of the 12V-15E and 15V-15E composite membranes: (a) porosity; (b) pore size. Data for individual VIPS (12V and 15V) and electrospun (15E) membranes are reminded.

The WCA and permeability of the composite membranes were then determined. The 15V-15E samples present a very hydrophobic nature, and the WCA did not change over the measurement duration. While CA is hydrophilic, the electrospinning technique leads to highly porous and rough surfaces, resulting in a very large WCA in air (135°). Similarly, the top surface of the 12V-15E membrane is also initially highly hydrophobic, but the WCA then drops after about 50 s of contact. Following the wetting results, we hypothesize that this phenomenon is a consequence of the more porous VIPS membrane underneath the nanofiber. Indeed, we highlighted that the VIPS membrane prepared from a 12 wt % polymer content was more easily wetted than that prepared from a 15 wt % polymer content. This difference in the wetting of the VIPS layer may then have influenced water absorption within the whole system, driving water in the composite membrane at different rates. Hence, a low polymer content in the VIPS layer would correlate with faster wetting of the composite membrane, while a high polymer content would correlate with the reverse.

Additionally, the trends for the WCA in air of the bottom surfaces of the composite membranes show a rapidly decreasing WCA. Electrospinning was performed directly on the bottom surfaces of the VIPS membranes. Therefore, the WCA of the top surface of the VIPS membrane (Figure 3a,b) should match the WCA of the bottom surface of the composite membrane (Figure 7a). The faster decrease in WCA observed in Figure 7a is rationalized by the fact that the top surface of the VIPS membrane was in tight contact with the rotating drum during the electrospinning process, which may have slightly flattened the surface, hence affecting its wettability.

**Figure 7:**
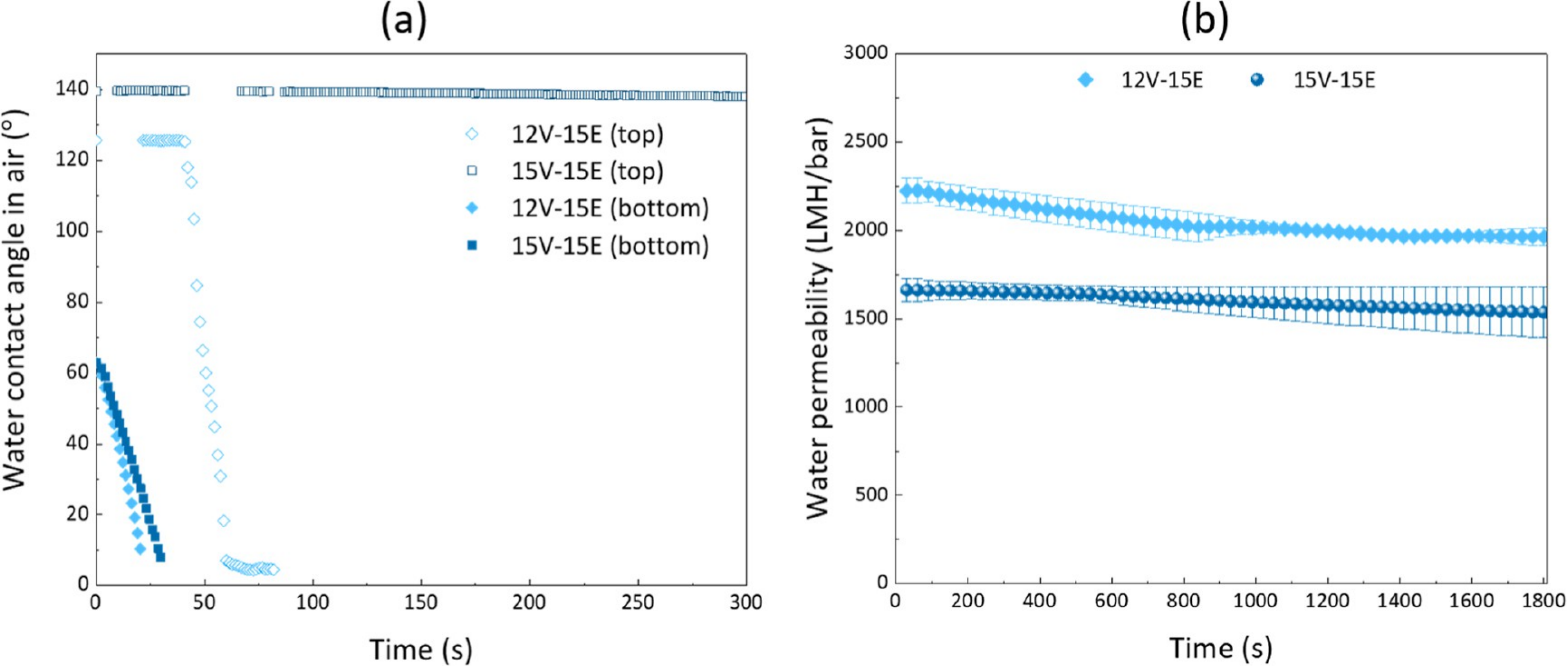
Hydrophilic properties of the composite membranes. (a) Water contact angle in air and (b) water permeability (tests conducted at 1 bar for 30 min).

Finally, the water permeability of the composite membranes was also measured, and the results depicted in Figure 7b reveal a notable high permeability for both membranes. It was measured to be about 2000 LMH/bar and 1700 LMH/bar for the 12V-15E and 15V-15E membranes, respectively. It is interesting to note again that the slightly larger pore size of the VIPS layer in 12V-15E, compared to 15V-15E, may contribute to easier water absorption and so faster water permeation through the membrane.

### Evaluating the Blood Compatibility of the Materials

The effects on the hemolysis activity of red blood cells and plasma clotting time were evaluated as biocompatibility indicators. In general, materials with a hemolysis rate lower than 2% are considered nonhemolytic.^38^ From the results presented in Figure 8a, it is clear that the contact of blood with the composite membranes does not trigger RBC lysis. As such, it can be concluded that they are nonhemolytic. In addition, the plasma clotting time of materials employed in blood-contacting devices can be evaluated.^39,40^ Long clotting times are an indication of the material hemocompatibility. In the condition of the test, the control material, a hydrogel made of sulfobetaine methacrylate (SBMA), displayed the longest plasma clotting time, as SBMA materials and derivatives have been widely investigated as potential suitable materials for blood-contacting interfaces.^40–42^ The composite membranes display a shorter clotting time (Figure 8b). This can be attributed to both the intrinsic nature of the material and to different film structuring (membrane vs hydrogel). However, it has to be noted that the clotting time was comparable to that obtained with microfiltration poly­(vinylidene fluoride) grafted with antifouling poly­(ethylene glycol) methacrylate brushes, which are reported to have a hemocompatible nature.^40^ In addition, compared to whole blood, proteins are concentrated in PPP facilitating clotting and, thus, decreasing the clotting time. Therefore, it was decided to advance with the filtration of blood products.

**Figure 8:**
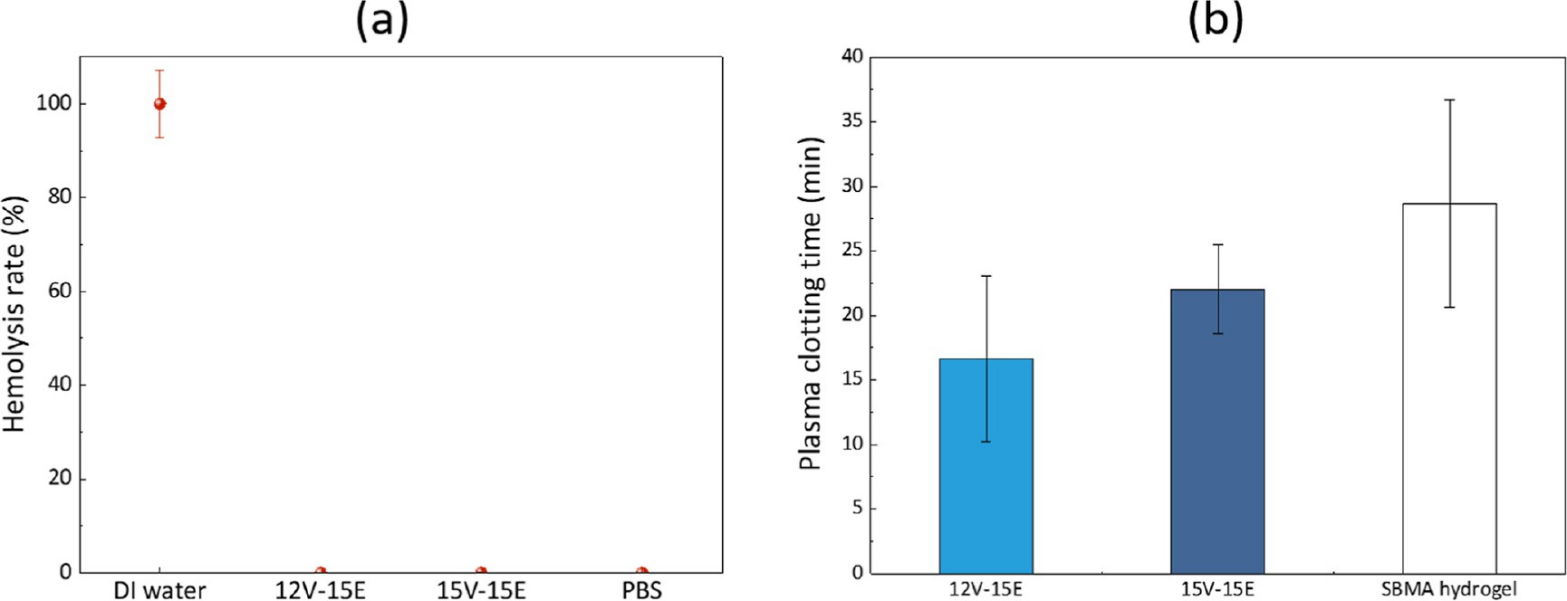
Assessment of the blood compatibility of the materials through their (a) hemolysis activity and (b) plasma clotting time.

### Removal of Cells from Blood Solutions

The primary objective of this study is to evaluate the effectiveness of the CA composite membrane in separating blood cells from plasma. Potential future applications could be for plasma separation and medical diagnostics^43^ where cells are removed to facilitate the detection of biomarkers in plasma. We exposed the membranes to solutions containing progressively higher concentrations of cells (platelet-poor vs platelet-rich plasma) or a more complex mixture of cells (platelet-rich plasma solutions vs solutions of whole blood), beginning with platelet-poor plasma (PPP) solution.

Despite its low cell concentration, as seen from the CBC analysis in Figure 9, filtration of PPP would still be needed to achieve cell-free plasma. However, compared to PRP and diluted or undiluted whole blood, PPP inherently contains much fewer cells. This rationale supports its selection as the feed for the initial assessment of the composite membranes’ efficiency. Filtration tests were carried out using both membranes 12V-15E and 15V-15E and all completed within 4 to 5 min. Slightly slower filtration times (and overall gravity-driven flux) were generally measured with sample 15V-15E, compared to 12V-15E, due to the smaller pore size of the VIPS layer (Table 1). The CBC analysis carried out on the permeates proved that the filtration with both composite membranes permitted us to remove entirely the platelets. It is also interesting to note the absence of major structural alteration of the membrane surface by comparing the SEM images shown in both Figure 9a,b (obtained after filtration) and Figure 5, suggesting the absence of platelet activation on the material surface. While the cell concentration in the feed solution may have not been high enough to effectively challenge the composite membranes and trigger biofouling by the platelets (confocal analysis did not permit us to observe any cells using PPP as the feed, unlike for PRP and diluted blood as seen later in this manuscript), this observation could also imply that the level of blood compatibility of the membranes was high enough for PPP filtration.

**Figure 9:**
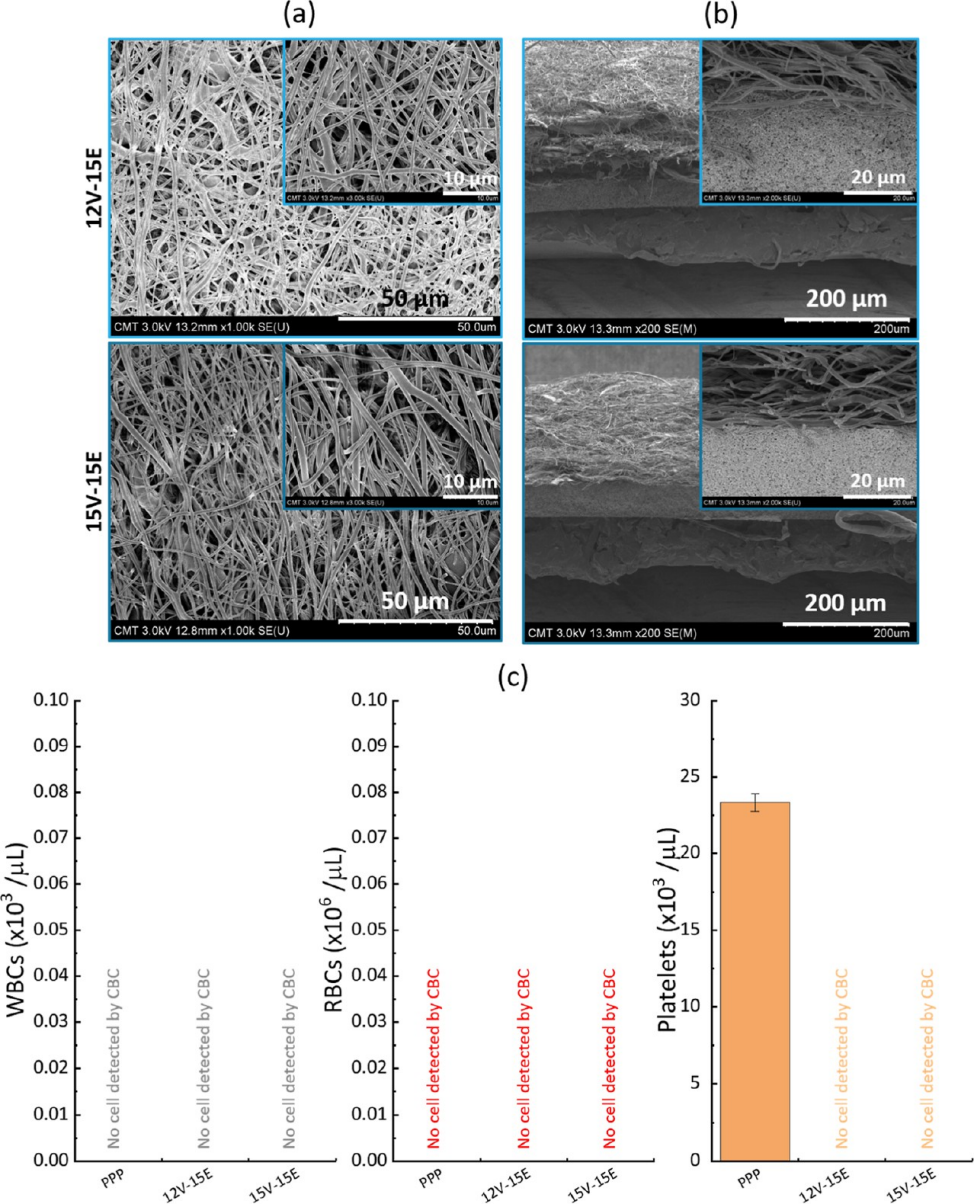
Filtration of a PPP solution with the composite membranes. (a) SEM images of the surfaces after filtration; (b) SEM images of the cross sections after filtration; (c) composition of the feed and permeate after filtration.

**Table 1:** Evaluation of the Overall Gravity-Driven Flux Associated with the Filtration of the Blood Solutions^a^

	12V-15E		15V-15E	
blood solution	time (min)	overall gravity-driven flux (L/m^2^ h)	time (min)	overall gravity-driven flux (L/m^2^ h)
PPP	4.1	897	4.6	800
PRP	4.3	855	4.8	766
10× diluted blood	5.5	669	5.5	669
5× diluted blood	N/A	N/A	7.7	478

a N/A: not assessed. Membrane diameter: 2.5 cm, feed volume: 3 mL, filtration mode: gravity-driven. The error on the flux (5 repetitions) is in the range 5–10%.

Platelet-rich plasma (PRP) contains a concentrated number of platelets but also RBCs and WBCs though their concentration is much lower than in whole blood (in the present case, the CBC analysis shown in Figure 10e revealed an RBCs concentration of about 0.013 ± 0.005 × 10^6^ cells/μL and a WBCs concentration of about 0.087 ± 0.006 × 10^3^ cells/μL). The membrane separation led to the formation of a dense layer on the surface of the membranes (arising from platelet activation and aggregation) and in the quasi-disappearance of the structure of the nanofibers, as clearly seen from the SEM images of Figure 10a, unlike that observed after PPP filtration, due to the much higher platelet count in PRP. Cells could then be readily visualized by confocal microscopy (Figure 10b). Considering the color-coded scale of the 3D confocal images in Figure 10d and the thickness of the membranes from the SEM images of the cross sections in Figure 10c, it seems that most cells were trapped in the electrospinning layer. However, cells were still detected in the VIPS membrane (yellow, orange, and red spots could be clearly observed, corresponding to deeper locations in the system). The SEM images in Figure S4 (magnified image of Figure 10d) also reveal that cells could penetrate inside the VIPS layer and aggregates, likely arising from platelet activation, were formed. Nevertheless, according to the CBC results presented in Figure 10e and comparing the composition of the feed (PRP) and of the permeate after the filtration, no cell could be detected when using either membrane 12V-15E or 15V-15E. Therefore, the present membranes can be combined with centrifugation to prepare cell-free plasma from PRP.

**Figure 10:**
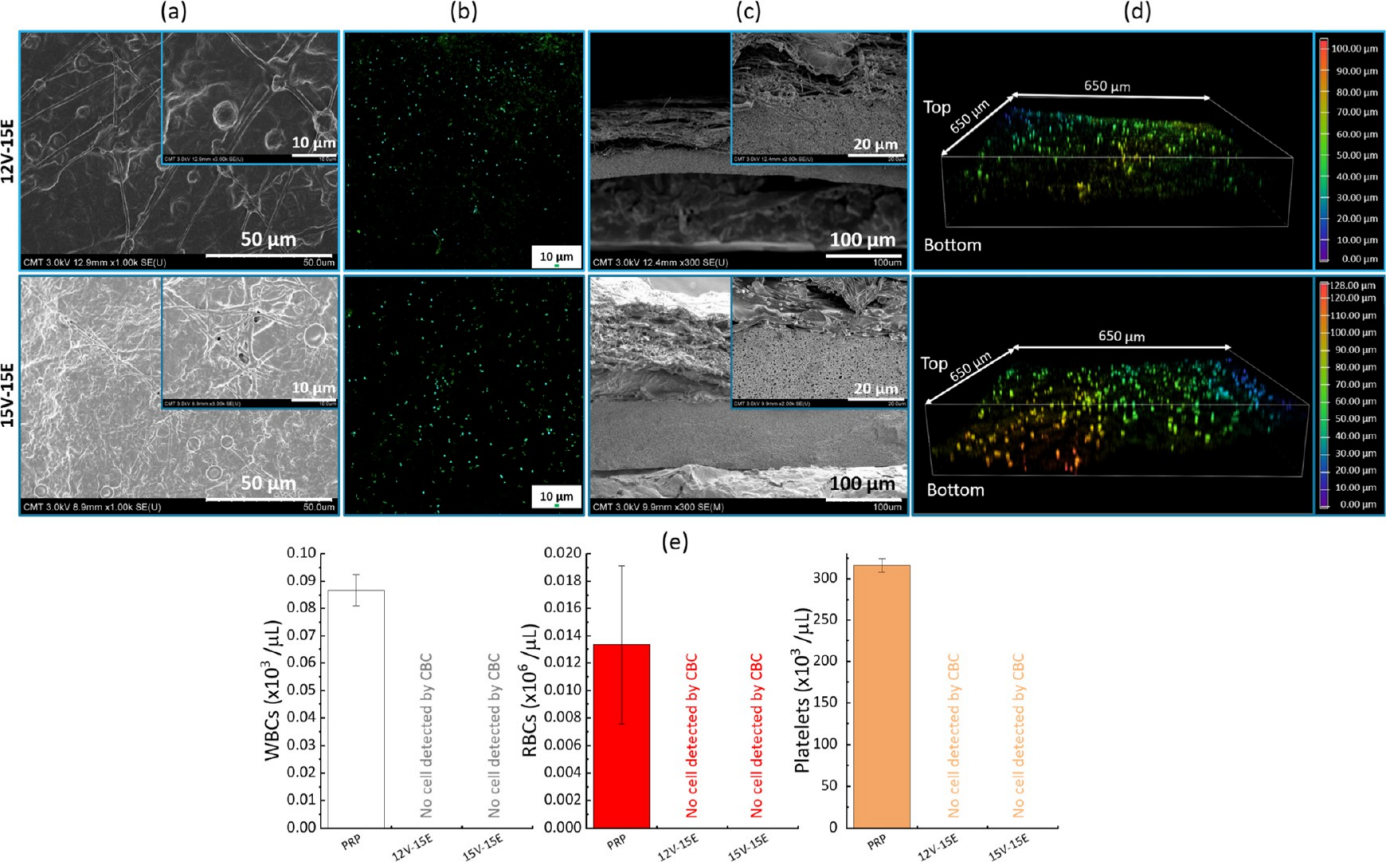
Filtration of a PRP solution with the composite membranes. (a) SEM images of the surfaces after filtration; (b) 2D confocal images of the membranes after filtration; (c) SEM images of the cross sections after filtration; (d) 3D confocal images of the membranes after filtration; (e) composition of the feed and permeate after filtration.

We then assessed the separation efficiency of the composite membranes with solutions of whole blood, focusing first on their performance in processing 10 times diluted whole blood. The decision to use a 10-fold dilution instead of undiluted whole blood was strategic, as the latter’s high viscosity made gravity-driven flow through the composite membrane challenging. While applying constant extra mechanical transmembrane pressure was a viable alternative, it posed the risk of inducing hemolysis, which is a complication we sought to avoid.

In Figure 11a–d is the SEM and confocal images of the surfaces after filtration with 12V-15E and 15V-15E membranes (completed within 5 to 6 min), whereas Figure 11e presents the complete blood count of the permeate. While membrane 12V-15E removes a large proportion of blood cells from the sample, having a removal of at least 95% on all blood cells, it still could not permit one to obtain cell-free plasma. In contrast, membrane 15V-15E permitted removal of all cells from the blood sample. This result arises from the ability of cells to deform and permeate through pores significantly smaller than their dimensions. They could still partially permeate through pores of diameter 0.41 μm (membrane 12V-15E) (Figure S3e). Conversely, the membrane with 0.36 μm pore size (15V-15E) seemed more effective at retaining all cellular components. This could also be assessed visually from the confocal images, as more cells were seen on images corresponding to 15V-15E than on images corresponding to 12V-15E. It is noteworthy that WBCs were more efficiently removed than RBCs, which, in turn, were more efficiently removed than platelets. This ranking correlated with the blood cells size.

**Figure 11:**
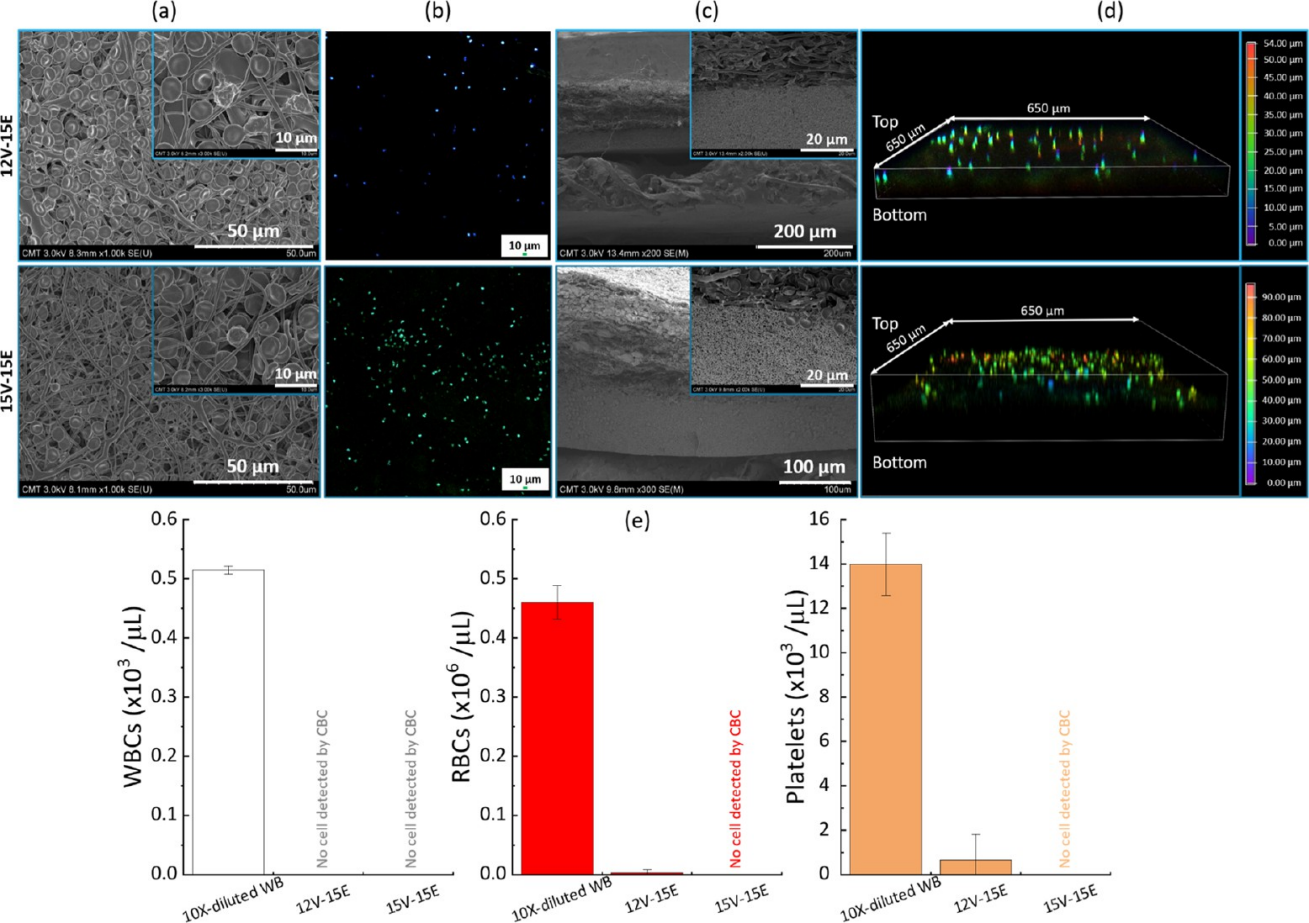
Filtration of a 10-fold dilution of whole blood using 12V-15E and 15V-15E membranes. (a) SEM images of the surfaces after filtration; (b) 2D confocal images of the membranes after filtration; (c) SEM images of the cross sections after filtration; (d) 3D confocal images of the membranes after filtration; (e) composition of the feed and permeate after filtration.

In Figure S5, we provide a magnified SEM image of the cross section of cells 15V-15E, where the closest cells to the bottom of the membranes are highlighted by red arrows. Despite significant dimension differences between the cells and the membrane average pore (diameter of RBCs is 7–8 μm with a thickness of 2 μm^44^), these cells are still discernible in close proximity to the membrane’s bottom surface. This SEM image could indeed represent the depth filtration mechanism. This is supported by the inherent deformability of cells permitting them to minimize their resistance to flow and navigate through very small capillaries.^45^ Moreover, membrane wetting and swelling by the blood solution may also have contributed to a slight enlargement of the pores, facilitating cells diffusion. As such, the penetration of blood cells deep inside the pores of the VIPS membrane is a possible scenario that should not be disregarded.

Another point worth mentioning is the difference between the surface structure of the membrane after filtration with PRP (Figure 10a) and diluted blood (Figure 11a). After filtration with PRP, the porous structure of the surface has almost entirely disappeared while with diluted blood, the fibers and pores are still detectable beneath the cells. This difference may be attributed to the tendency of platelets to undergo aggregation and activation in PRP compared to diluted blood. Würtz et al. reported a correlation between platelet count and aggregation in patients with coronary disease.^46,47^ Assuming that the correlation holds true in the present case, the higher concentration in platelets in PRP, compared with whole blood and diluted whole blood, facilitated their activation, resulting in the formation of a dense layer of fibrin and platelets.

As positive results were obtained with a 10-fold dilution of blood, tests were then performed using 5-fold diluted whole blood, and the results are presented in Figure 12. Solely the membrane 15V-15E was utilized for this test, as the membrane 12V-15E partially failed at providing a cell-free permeate with the 10-fold dilution of blood. The analysis of the permeate shows that the membrane could effectively retain all blood cells detectable by the CBC instrument. Again, the SEM images reveal that the surface of the membrane was covered with numerous cells. Due to the higher cell concentration than in the 10-fold dilution, it is reasonable to assume that fouling also facilitated the cell retention. Importantly, there was almost no hemolysis during the filtration, as the hemolytic activity on the permeate was measured to be <0.04% (±0.1%). This finding carries significance in the context of developing a membrane capable of producing cell-free plasma from blood solutions using a single asymmetric membrane, as the literature often suggests the necessity of employing multiple membranes in cell removal from whole blood such as leukoreduction,^22,48^ to ensure complete cell removal. However, despite the promising result in terms of membrane functionality, the filtration of pure whole blood within a reasonable time frame was hardly achievable under the test conditions, as it took more than 20 min (same order of magnitude as the plasma clotting time). This indicates the need for further improvements to our design.

**Figure 12:**
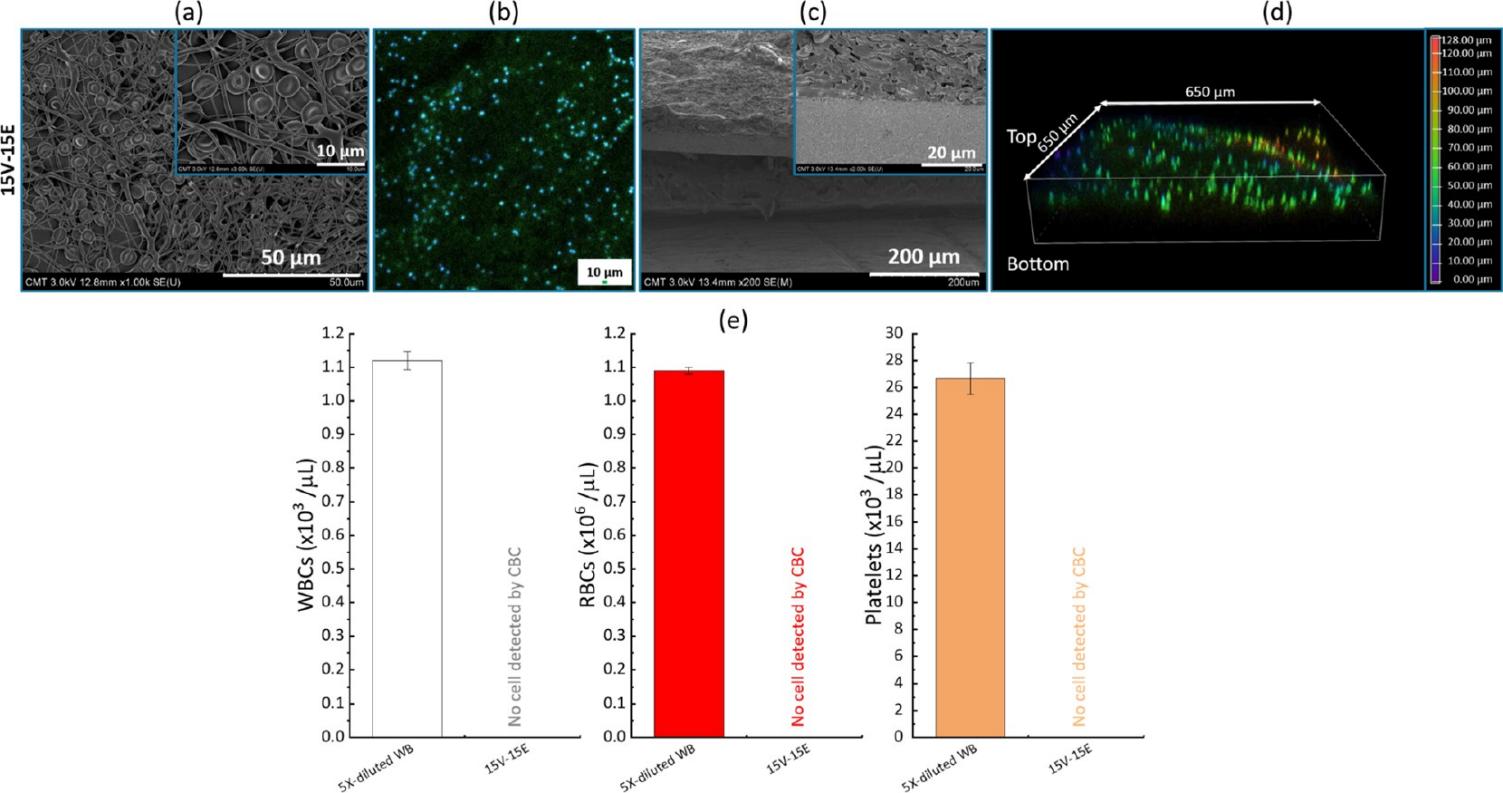
Filtration of a 5-fold dilution of whole blood using the 15V-15E membrane. (a) SEM images of the surfaces after filtration; (b) 2D confocal images of the membranes after filtration; (c) SEM images of the cross sections after filtration; (d) 3D confocal images of the membranes after filtration; (e) composition of the feed and permeate after filtration.

### Determination of the Total Plasma Protein after Filtration

Biomarkers found in blood that are used for the detection of specific diseases are usually proteins. It would be ideal to have minimal reduction in the plasma protein concentration after the filtration process in order for the cell-free plasma to be viable for the detection of biomarkers. Thus, the concentration of the plasma proteins after filtration was verified. The normal clinical concentration of the total plasma protein of a healthy individual is in the range of 62,000–78,000 μg/mL.^49^ Figure 13 shows the total plasma protein concentration of 10-fold and 5-fold blood dilution and their filtrates after the filtration with the 15V-15E membrane. It can be observed that the protein concentration of the filtrate of the 5-fold diluted blood was greatly reduced compared to that of the filtrate of the 10-fold diluted blood. This could be rationalized by observing the cross sections of the 15V-15E membrane after filtration with 10-fold and 5-fold diluted whole blood (Figures 11c and 12c). It can be seen that a much more severe fouling was evidenced by the cake formation in the electrospun layer. The 15V-15E membrane used for the filtration of the 10-fold diluted whole blood shows numerous red blood cells in the electrospun layer while the one used for the filtration of the 5-fold diluted whole blood clearly shows aggregates similar to the one observed during the filtration of PRP (Figure 10a). This observation led us to conclude that at higher concentrations of whole blood, there is significantly more aggregation or thrombus formation which uses up protein in the blood. A specific example would be fibrinogen, a protein dissolved in blood, which is converted to fibrin (an insoluble protein) during clot formation.^50^

**Figure 13:**
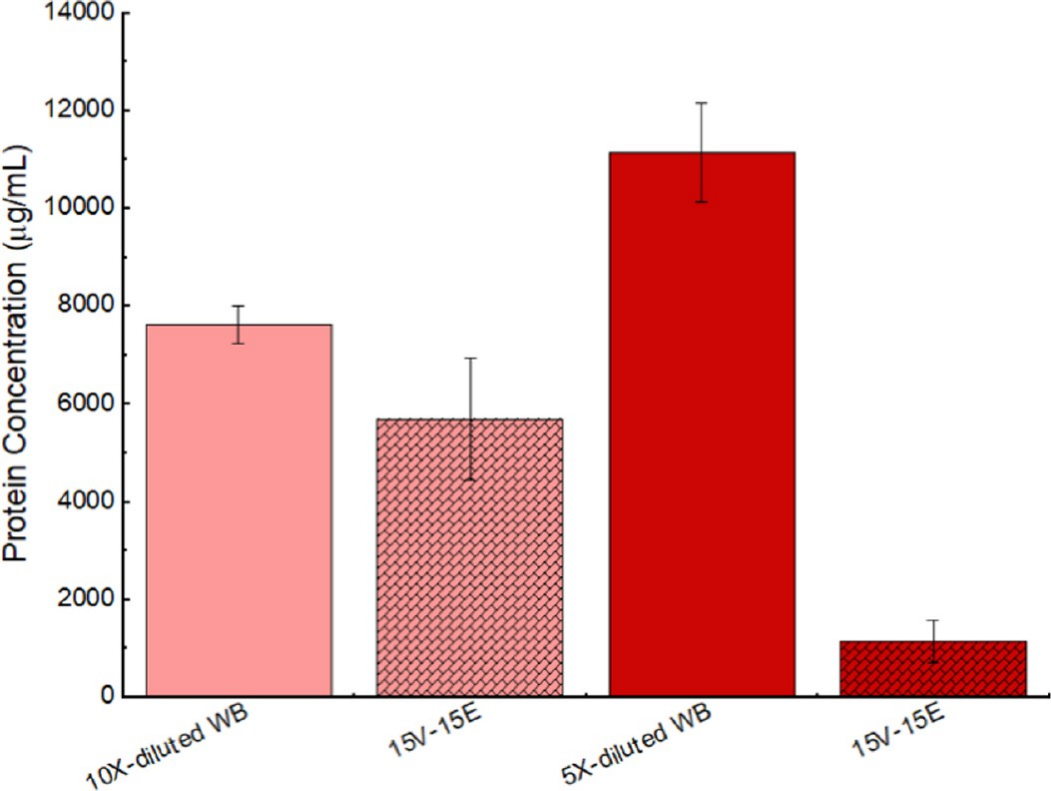
Total plasma protein concentration measured using Bradford assay for 10-fold and 5-fold diluted whole blood and their filtrates with the 15V-15E membrane.

Other biomarkers, such as glucose, total cholesterol, and triglycerides, were also evaluated and are presented in Figure S6. Additional samples such as whole blood and PPP were also tested as references. Comparing the results of 5×-diluted whole blood before and after filtration with the 15V-15E membrane shows that there is a greater reduction in glucose levels compared to total cholesterol and triglycerides. Glucose levels were reduced by 47.61% (from 118.3 mg/dL to 62.0 mg/dL) while total cholesterol was only reduced by 6.25% (from 10.6 mg/dL to 10.0 mg/dL) and triglycerides by 35.23% (from 14.7 mg/dL to 9.5 mg/dL). The losses with these biomarkers are lower compared to the loss in total protein after filtration. This may indicate that the participation of these biomarkers in the blood coagulation cascade is minimal. Another possible explanation for these losses relates to the hydrophilicity of the VIPS layer. Since glucose is a water-soluble molecule, unlike cholesterol and triglycerides, the membrane’s water uptake could contribute to a more significant loss of glucose.

### Asymmetric vs Symmetric Structure Performances during the Filtration of Diluted Blood (5×)

The composite membrane is designed to gradually remove cells during the filtration process and enable flow. However, one could reasonably assume that the VIPS layer alone might be capable of removing all cells. To validate the need for the composite structure, filtration of the 5-fold dilution of whole blood was also carried out with membrane 15V, containing the VIPS layer only, and the filtration outcome was compared to that obtained after filtration with membrane 15V-15E. The following conclusions can be drawn from the results presented in Figure 14. First, there was no blood permeation when using the VIPS membrane alone (Figure 14a), while permeation occurred with the asymmetric membrane. This visual observation alone justifies the need for a composite structure. Cells can gradually penetrate within the electrospun layer and are progressively retained by depth filtration over its entire cross section (Figure 14d). Thus, when the blood solution reaches the VIPS layer, its cell concentration has decreased greatly, facilitating permeation through the smaller pores. In contrast, when the VIPS membrane is directly exposed to the blood solution, the larger cell concentration results in quick cake formation on the surface. It is seen from the SEM images that the formation of a cake layer after platelet adhesion blocked the porous structure of the membrane (Figure 14c). Oppositely, the structure of the fibers is still seen after filtration using the asymmetric membrane because they possess much larger pores. As the pores of the VIPS membrane disappeared, the surface became smoother, which also likely explains the whitish color of the membranes after the filtration process as red blood cells could be readily washed off (Figure 14b). Oppositely, the asymmetric membrane appeared red because of the numerous RBCs trapped inside the fibrous layer. This visual observation is also consistent with the type of cells observed by SEM: numerous RBCs were seen on the surface after filtration with membranes 15V-15E, while their characteristic biconcave shape could hardly be detected on the surface of membrane 15V (instead, platelets were mostly detected). Therefore, to allow flow by gradually removing blood cells without cell lysis (we emphasized in the previous section the low hemolysis after filtration with membrane 15V-15E), the asymmetric structure with very large pores on the top surfaces and gradually smaller pores in the deeper layer is needed.

**Figure 14:**
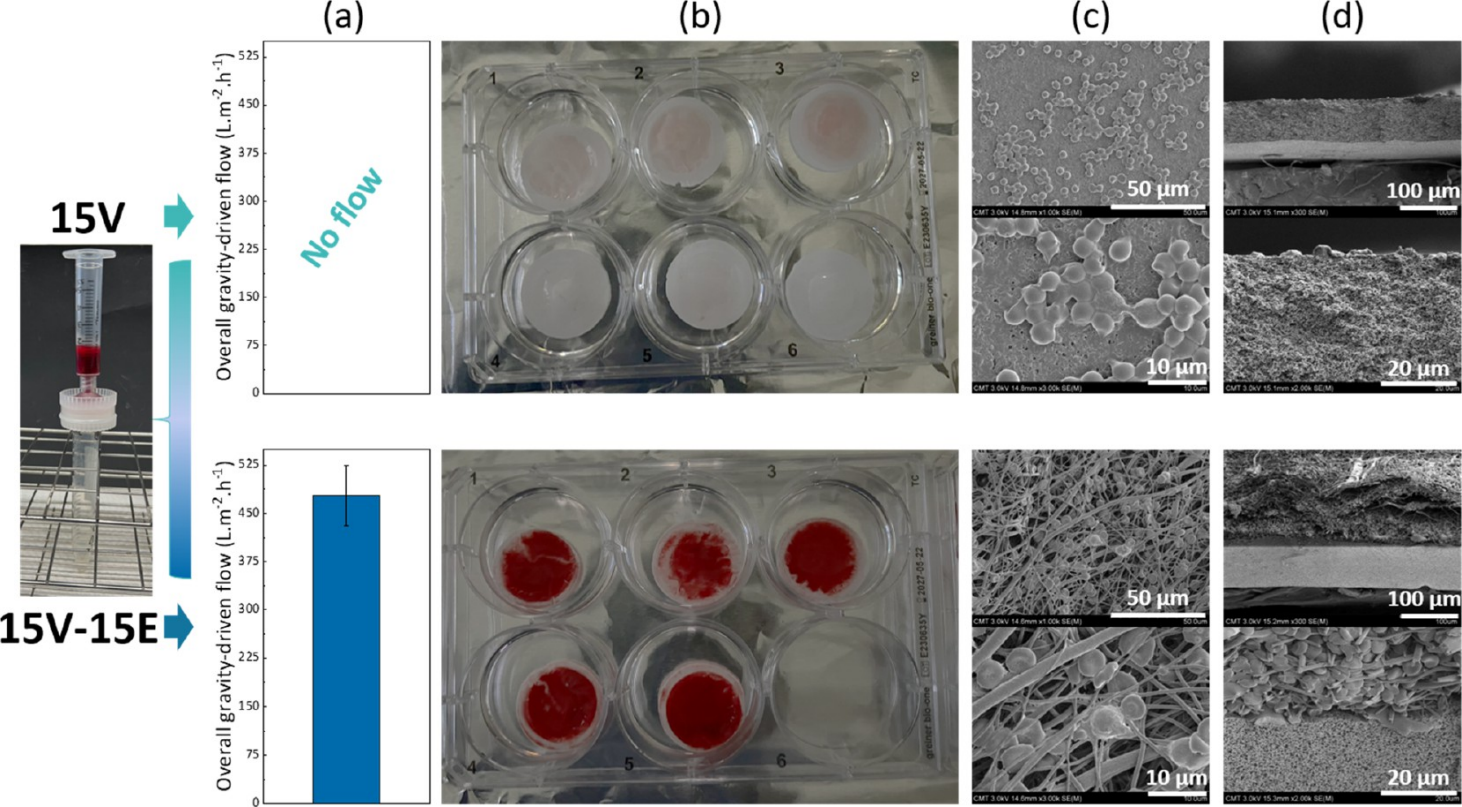
Comparison of the filtration outcome using the 15V membrane and 15V-15E membrane. (a) Overall gravity-driven filtration flux; (b) photographs of the membranes after the filtration; (c) SEM images of the surfaces after the filtration; (d) SEM images of the cross sections after the filtration.

### Discussion—Some Potential Directions to Improve the Membrane Design

The results obtained in the frame of this study show promising separation of blood cells from plasma utilizing diverse blood-derived solutions. However, it remains challenging, with the system at play, to separate cells from undiluted whole blood without applying extra mechanical pressure that would trigger cell lysis. At best with the current VIPS-electrospinning composite CA membrane, separation of blood cells from plasma could only be performed with a 5-fold dilution in a reasonable duration (Table 1). Therefore, improvements could be made to the material system and to the membrane design as well and concern the following aspects.

- Cellulose acetate was chosen for its good film and fiber-forming property^51^ and its relative intrinsic hydrophilicity.^52^ But the level of hydrophilicity can be tuned by adjusting the acetylation degree. Zhou et al. reported that the WCA of spin-coated CA films could vary from 0° to 132° by increasing the degree of substitution of the material.^53^ While this may be at the expense of the mechanical stability, this direction deserves to be explored. In addition, the work of Liu and Tang, who utilized several CAs with a similar acetyl content but a varying molecular weight, evidenced improved hydrophilicity of the nanofibers with the molecular weight.^28^ More specifically, these authors reported that the WCA that could be reduced from 81° to about 40° for similar solvent system, polymer concentration, and spinning parameters.
- The grafting/coating of zwitterionic materials onto CA may permit further improvement of membrane performances. For instance, Liu et al. developed zwitterionic cellulose acetate membranes to mitigate fouling of ultrafiltration membranes during the filtration of a protein solution^54^ and revealed a sharp increase improvement of the membrane surface hydrophilicity and water permeability. Moreover, a recent study starred the utilization of a zwitterionic trimethoxysilane compound for the modification of CA membranes applied in desalination.^55^ Zwitterionic materials are known for their excellent hydration behavior,^56^ and CA still contains numerous reactive sites (hydroxyl pendent groups) which offers multiple possibilities for surface functionalization with amine compounds^54^ or derivatives of oxirane.^57^ For instance, in the study of Liu et al., the Schiff base reaction involving the oxidized cellulose acetate was then followed by a sulfonation reaction.^54^ But a material containing both a zwitterionic moiety and an amine reactive group (for example, obtained by reaction between 3-(dimethylamino)­propylamine and 1,3-propane sultone^58^) could be tested as well. Regardless of the synthesis route, the multiple possibilities offered by the reactivity of CA could benefit the membrane design. Moreover, the functionalization of the CA membrane with zwitterionic materials, sulfobetaine-based,^42,59^ carboxybetaine-based,^41,60^ or phosphobetaine-based,^61^ would improve the blood compatibility of the filter. We have mentioned in the previous section the long duration for whole blood separation, in the range of the clotting time for PPP. Surely, proteins are concentrated in PPP, facilitating clotting and so decreasing the clotting time. Yet, a too long contact of whole blood with the membrane may trigger clotting on pure CA. Adding some zwitterionic material would not only improve the hydrophilicity and enhance blood flow but also improve the hemocompatibility, permitting longer contact of blood with the material and perhaps the filtration of undiluted human whole blood. A recent study showed the positive effect of a zwitterionic material (polycarboxylate polyurethane) on the membrane performance (fouling resistance, flow) during the separation of plasma and porcine whole blood.^62^ Although a pressure gradient was applied with an external pump and nonhuman blood used, this approach did demonstrate the benefit of the zwitterionic modification.

Linked to the potential benefits of the zwitterionic modification of the membrane, we observed that the structure disappeared due to platelet aggregation mostly in two specific instances: when PRP was filtered and when the VIPS membrane was used alone. It is important to note that the platelet concentration in PRP is significantly higher than that in whole blood. In fact, when diluted blood is filtered using the appropriate composite membrane (as opposed to the VIPS membrane), the structure can still be detected, potentially indicating reduced platelet aggregation. This may be because the composite structure design enabled the gradual removal of cells through the large pores of the electrospun layer, reduced stress, and prevented the accumulation of platelets at the same location. Nevertheless, dedicated tests would be needed to ascertain the effect of the membrane structure and roughness on platelet aggregation and activation, in light of previous literature emphasizing the role of the surface hydrophobicity and roughness toward platelet activation.^63,64^ Additionally, it will then be interesting to assess the benefit of zwitterionic modification on this phenomenon.

- Aulin et al. showed that crystalline ordering and the mesostructure of crystalline films of cellulose formed by spin-coating could affect their swelling.^65^ In addition, Wu et al. mentioned that the dissolution of CA in an acetone/DMAc mixture enabled recrystallization, resulting in an increase of the crystallinity of the film compared to the original polymer.^66^ The same authors commented on the positive effect of the stretching of the macromolecule during the spinning process on the final film’s crystallinity. Thus, it may be interesting to investigate the potential interplay between solvent composition (or more generally spinning process parameters), crystallinity of the nanofibers, and wetting.
- Adjusting further the process parameter for faster wetting is also an important direction that our group intends to investigate. Structuring of the fibrous mat (size of the fibers and organization controlling the porosity) is well-known to influence wetting phenomena. However, taking into account the “roughness” of each individual fiber may also be a parameter to consider for better wetting of the CA layer. For example, the study of Lasprilla-Botero et al. reveals that different evaporation rates of the solvent/cosolvent system during the electrospinning process of polyimide nanofibers could generate small pits.^67^ In contrast, nanofibers formed using one single solvent were smoother. It caused the membrane formed using two solvents to display higher hydrophobicity than that formed using one single solvent. In this study, we based our choice of the solvent system on a previous work,^28^ as we focused mostly on the obtaining of regular bead-free nanofibers. Nevertheless, it could be interesting to take into account the difference in solvent evaporation rates during the spinning process, and its potential effect on surface wetting via the surface roughness, and so to try to design CA nanofibers using one single solvent.
- Exploring other methods for the formation of both the top and bottom layers should not be overlooked. One such method could be spin-coating. Cobo et al. produced a thin film of cellulose acetate whose pore size fell in the microfiltration range^68^ and highlighted correlations between pore size, polymer content, and polymer molecular weight. Another interesting aspect is that very thin films can be formed by this method,^69^ with relatively low surface roughness.^68^ These aspects would benefit wetting by blood and flow, and as such, spin-coating may also be a valuable method to account for in the membrane design, of the upper layer as a replacement of the electrospinning membrane if the pore size can be large enough, or of the lower layer as an alternative for the VIPS membrane.

One could also argue that membrane structures obtained by NIPS are inherently asymmetric and, as such, could provide an effective platform for blood cell removal. Nevertheless, we failed at obtaining porous top and bottom surfaces as shown in Figure 2 for an exposure to vapors set to 0 min (i.e., direct NIPS process). Without a VIPS step, a skin layer is obtained on top, which was also observed earlier.^8^ It results in the water permeability of CA membranes or CA-derived membranes prepared by NIPS being very low,^70^ compared to that measured with the composite membrane (Figure 7b). This low permeability poses a challenge when dealing with blood, which is more viscous than water, especially in a gravity-driven setting. However, it may be possible to create skin-free asymmetric CA membranes by NIPS with also a porous bottom surface by testing solvent/cosolvent combinations, changing the nonsolvent composition, or using pore-forming additives. This direction can be explored.

## Conclusions

In this work, composite membranes were prepared by electrospinning a cellulose acetate layer on a VIPS membrane made of the same material in view of creating an asymmetric structure and testing its applicability in the filtration of blood products. The first part of this study consisted of optimizing the VIPS layer on the one hand and the electrospinning layer on the other hand. Then, the composite material was shown to exhibit high porosity (83–84%). Its mean pore size (0.37–0.42 μm) was dependent on that of the VIPS layer, which has smaller pores than the electrospinning layer. The composite membranes’ wetting behavior was strongly associated with its structure: the top layer, formed of nanofibers, was much more hydrophobic than the bottom layer, corresponding to the denser surface of the VIPS membranes. Nevertheless, the CA membrane still displayed hemocompatibility signs (no hemolytic activity and a relatively long clotting time) and enabled the filtration of blood products. The results of the CBC analysis highlighted that the composite membrane 15V-15E (15 wt % CA in the dope solution used to form both layers) was able to remove entirely all cells from different blood-derived solutions including PPP, PRP, a 10-fold dilution of whole blood, and a 5-fold dilution of whole blood. Oppositely, there was no flow with the VIPS membrane alone. Despite the need for more adjustments to the membrane system, the results of this study provide valuable insights into the formation of biomedical membranes for blood cell removal that could be potentially useful for improved medical diagnosis.

## Supplementary Material



## Notes

SEM images of VIPS membranes including pore size analysis, SEM images after filtration of PRP, and SEM images after filtration of diluted blood (DOCX)
