# Supplementary material for: Assessment of the Blood Separation Performances of Asymmetric Cellulose Acetate Membranes Prepared through Combined Vapor-Induced Phase Separation and Electrospinning
Source: ACS Appl Mater Interfaces. 2025 May 20;17(22):31923–40. doi: 10.1021/acsami.5c05283 (PMC12147082; doi:10.1021/acsami.5c05283)
Supplement: Supplementary file 1 [file am5c05283_si_001.docx]

# Supporting Information

Assessment of the Blood Separation Performances of Asymmetric Cellulose Acetate Membranes Prepared through Combined Vapor-Induced Phase Separation and Electrospinning

*Gian Vincent Dizon, Yu-Jen Huang, Fang-Cheng Lin, Irish Valerie Maggay, Yung Chang,* Antoine Venault***

R&D Center for Membrane Technology and Department of Chemical Engineering, Chung Yuan Christian University, Chung-Li 32023, Taiwan (R.O.C)

Corresponding authors: *[ychang@cycu.edu.tw](mailto:ycahng@cycu.edu.tw) (Y. Chang), **[avenault@cycu.edu.tw](mailto:avenault@cycu.edu.tw) (A. Venault)

Complementary SEM images of VIPS membranes

Figure S1 gathers SEM images corresponding to VIPS membranes prepared by fixing the exposure time to non-solvent vapors from 0 to 20 min (other parameters including the polymer concentration, 15 wt.%, or the relative humidity inside the chamber, 70%, were kept constant). It is particularly noticeable that for 10 min, the top surface still is dense, while small pores can be detected if the exposure time is set to 20 min.


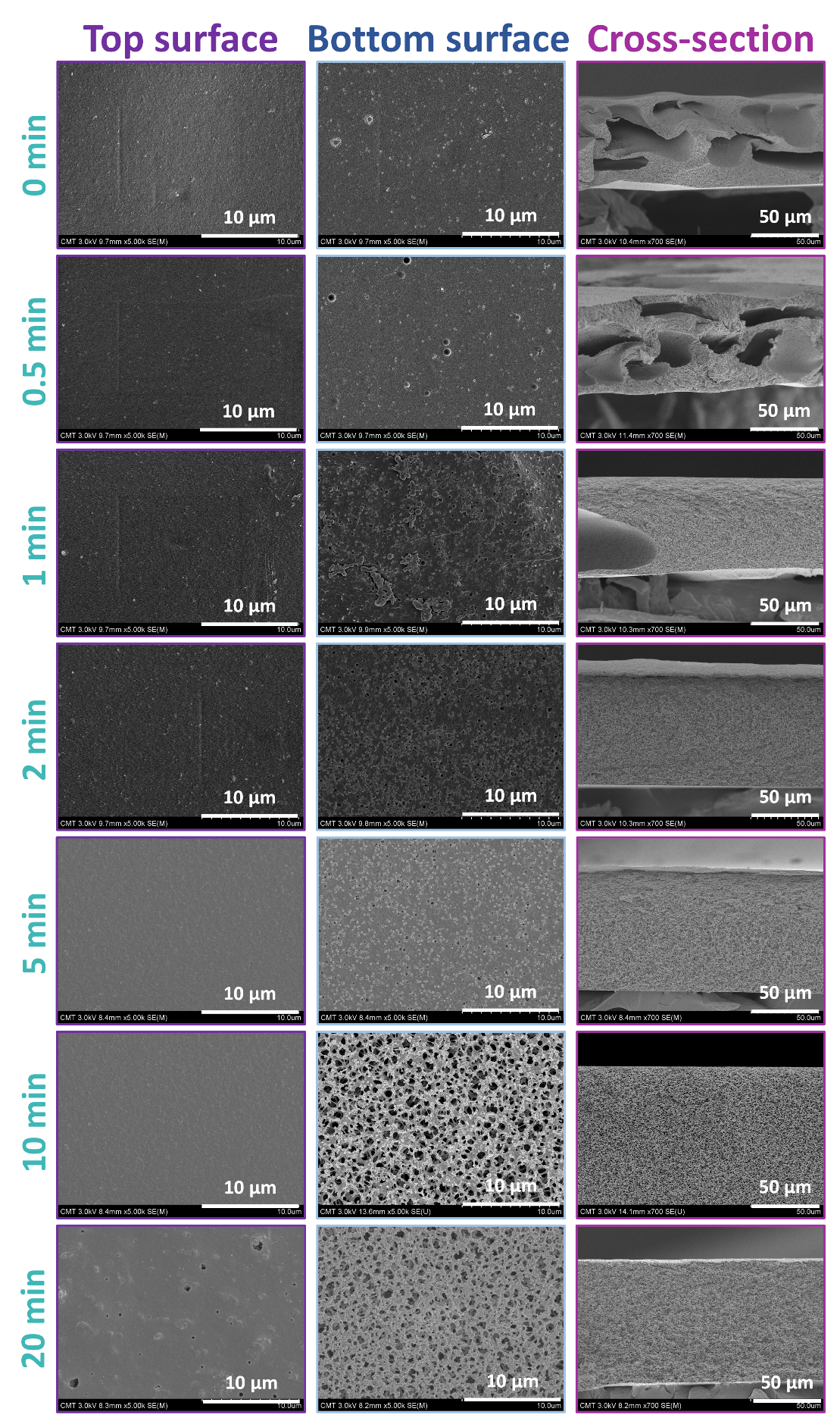


**Figure S1.** Effect of the exposure time to non-solvent vapors on the morphology of the VIPS membranes. Top and bottom images have a magnification of x5000 while cross section images are x700.

Effect of the casting solution concentration on the morphology of the membranes

Figure S2 shows the SEM images of the cellulose acetate membrane at different concentrations of the casting solution. Morphologies of the top, bottom, and the cross-section are shown. It is observed that the top surfaces of the membranes are generally dense and a change in the pore size is only observed in the morphologies of the bottom part of the membranes.


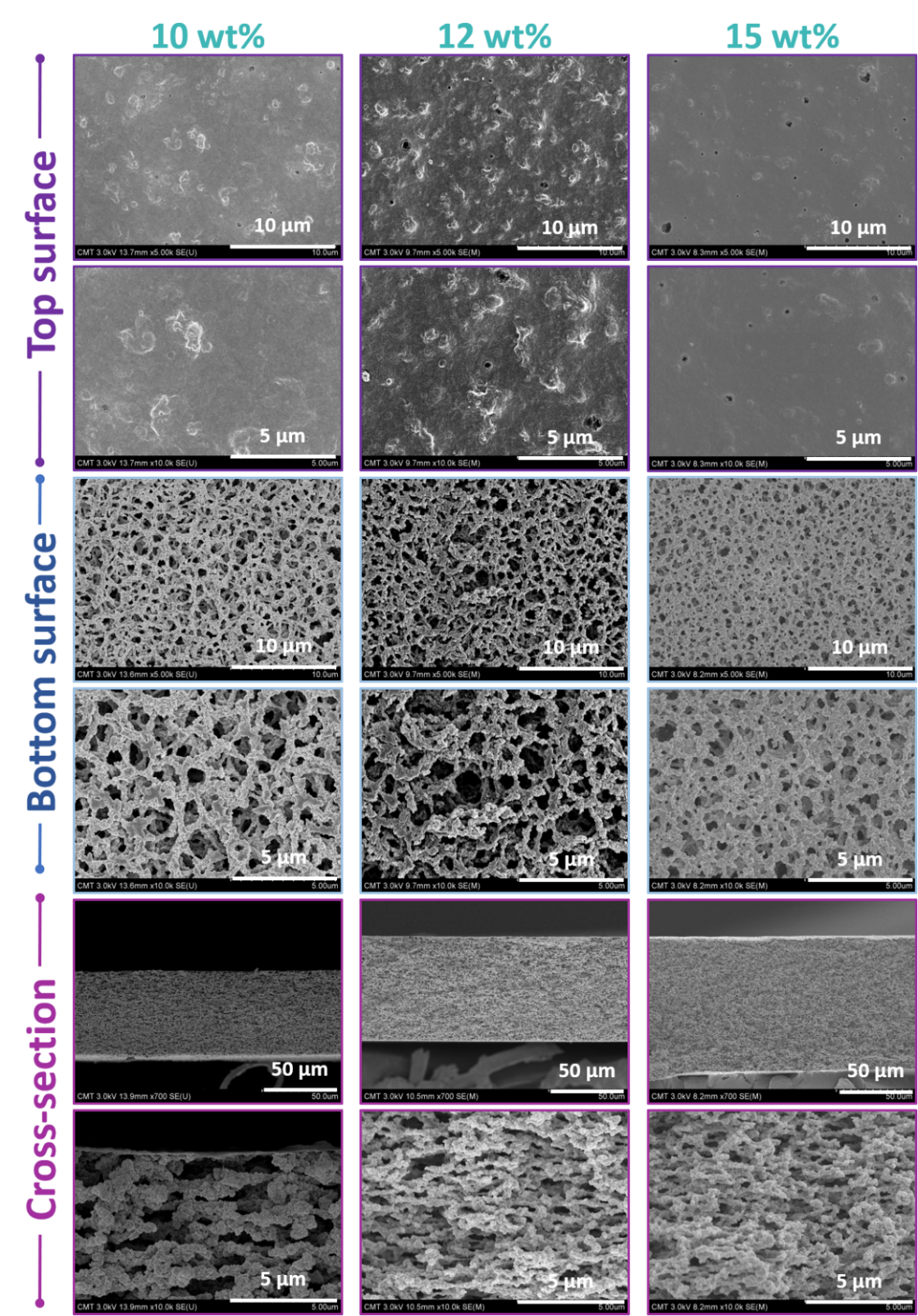


**Figure S2**. Effect of the CA concentration in the casting solution on the morphology of the VIPS membranes (exposure time to vapors: 20 min, relative humidity: 70%).

Pore size analysis based from the SEM images

Figure S3 shows the pore size distribution of the top and bottom surfaces of the membranes which was analyzed from their SEM images using ImageJ. A very minimal decrease in the mean bulk pore size is observed from the increase of the concentration of the casting solution from 12 wt% to 15 wt%.


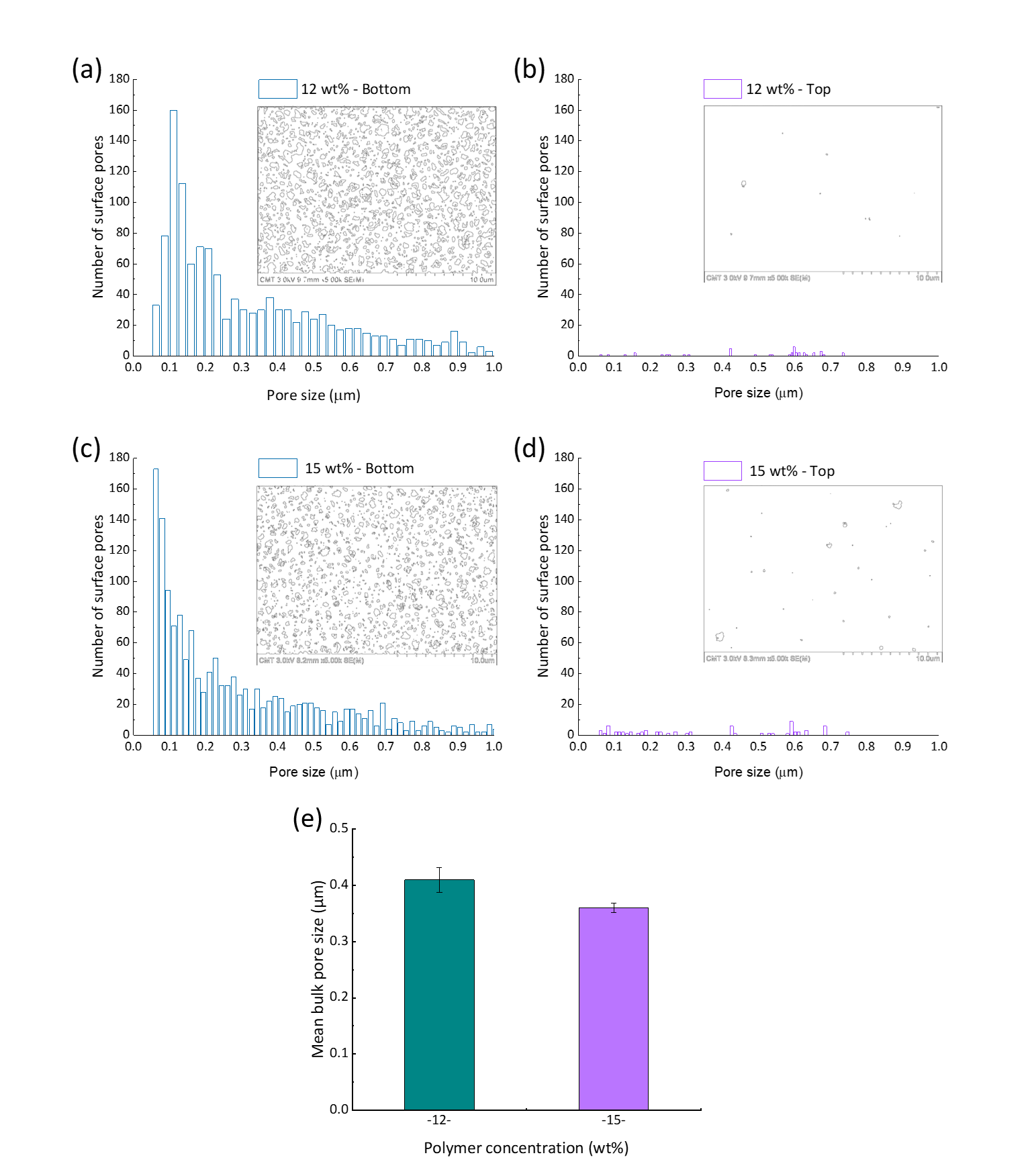


**Figure S3**. Pore size analyses of the VIPS membranes prepared after 20 min exposure to water vapors. (a), (c) Bottom surface pore size, (b), (d) Top surface pore size, (e) Mean bulk pore size.

SEM images after filtration of PRP

Figure S4 presents the cross-section of 12V-15E membrane after filtration of PRP. The brown arrows highlight aggregates in the cross-section of the VIPS layer. They would arise from platelet attachment and activation. Hence, platelets could still partially penetrate the VIPS membrane, although the electrospun layer was able to “stop” most of these cells as seen from the disappearance of the fibrous structures on top of the composite membrane.

**Figure S4.** SEM image of the cross-section of 12V-15E membrane after filtration of PRP. The brown arrows point at aggregates in the cross-section of the VIPS layer.

SEM images after filtration of diluted blood

Figure S5 presents the cross-section of 15V-15E membrane after filtration of diluted whole blood (10-fold dilution). The red arrows highlight the position of cells (RBCs) detected close to the bottom surface. Despite having significantly larger dimensions than those of the pores, the cells may have still penetrated deep inside the filter, due to their elasticity.


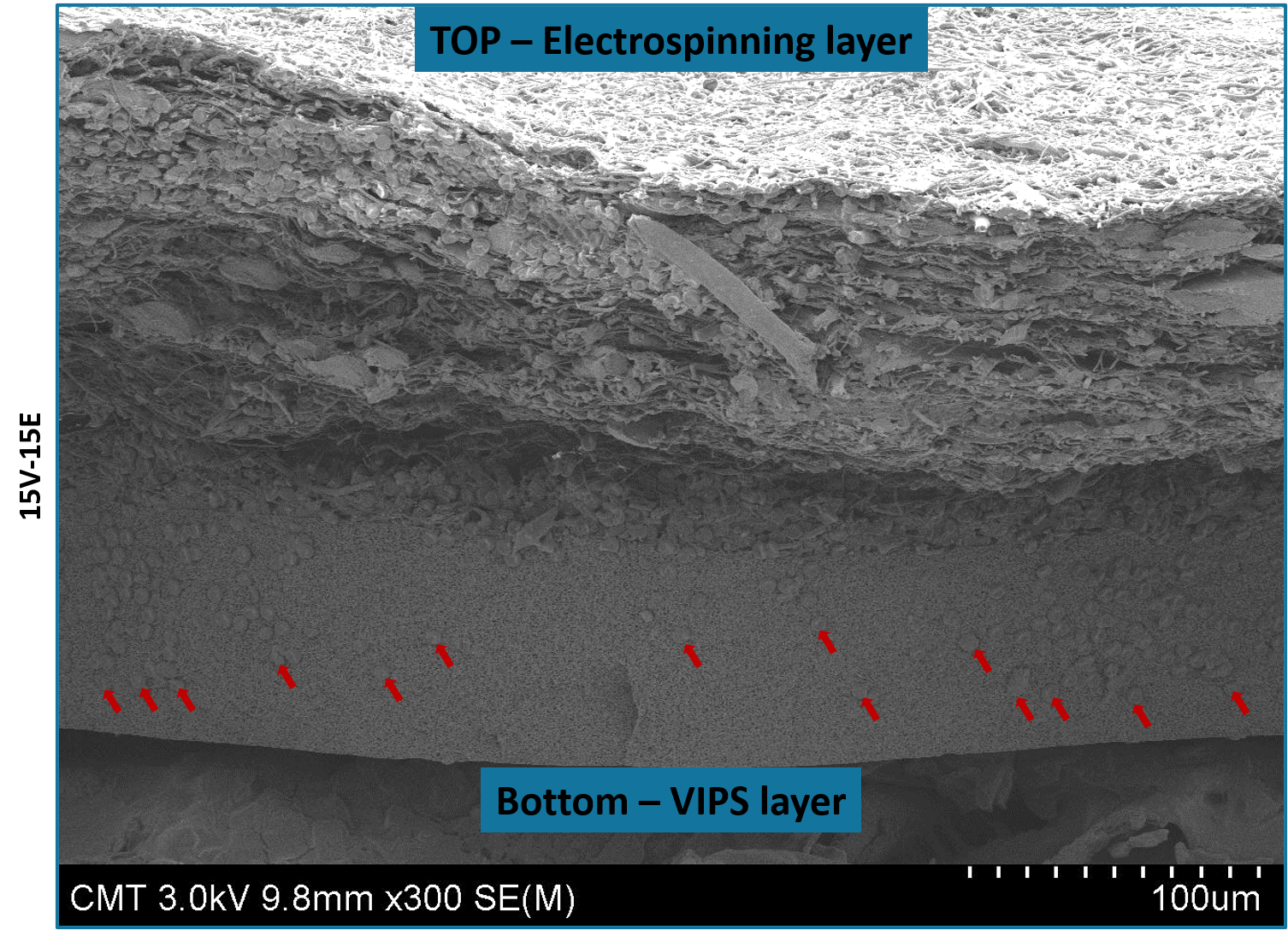


**Figure S5**. SEM image of the cross-section of 15V-15E membrane after filtration of diluted whole blood. The red arrows point at cells close to the bottom surface of the composite membrane.

Other biomarkers in serum

Figure S6 shows the glucose, total cholesterol, and triglyceride levels of different samples. Results for whole blood and PPP show no significant difference, meaning centrifugation does not affect these biomarkers. These biomarkers are small enough to be suspended in the plasma even after centrifugation. The results before and after filtration of 5X-diluted WB shows minimal reduction.


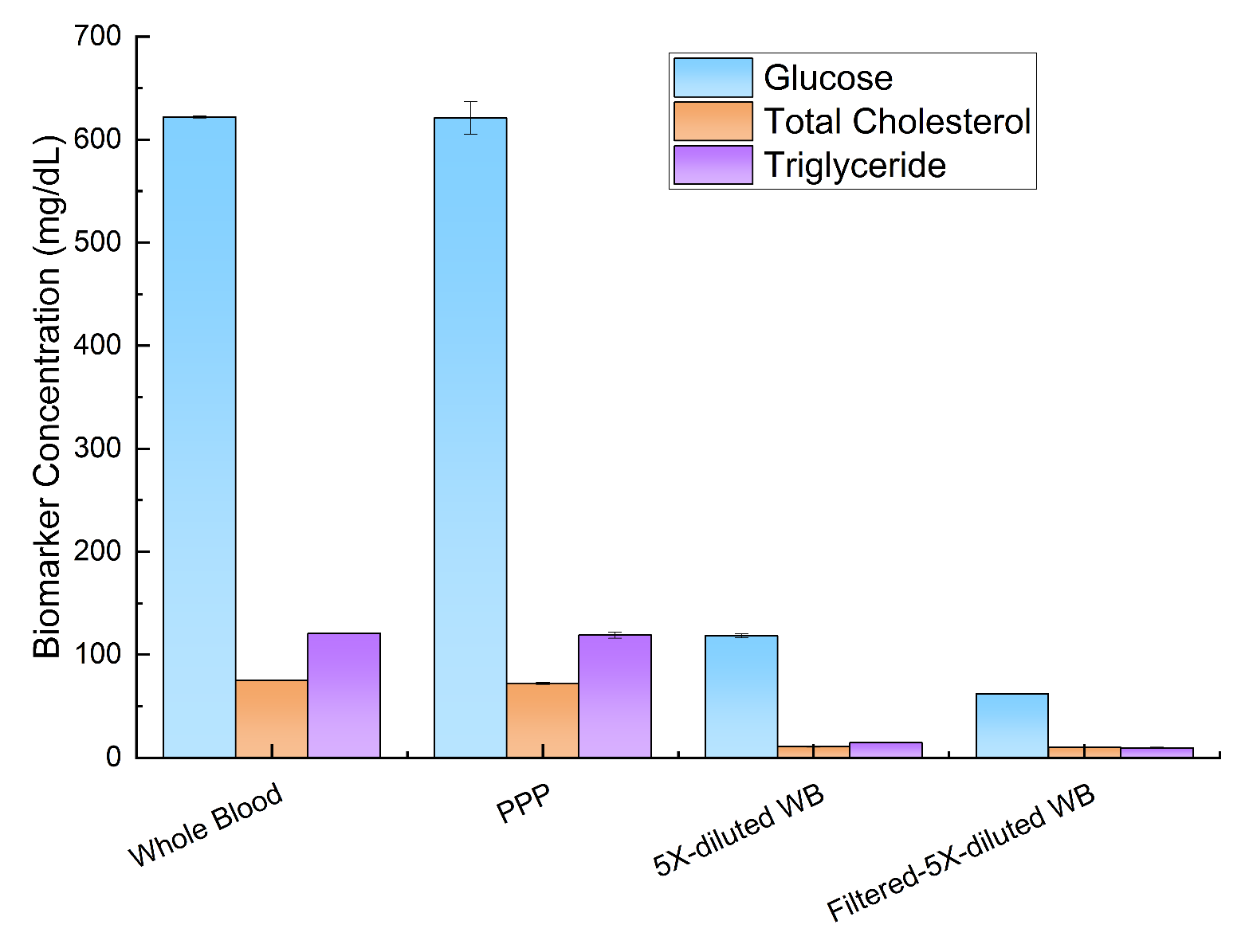


**Figure S6**. Other biomarkers such as glucose, total cholesterol, and triglycerides that were evaluated before and after filtration with the 15V-15E membrane.
